# The NIN transcription factor coordinates CEP and CLE signaling peptides that regulate nodulation antagonistically

**DOI:** 10.1038/s41467-020-16968-1

**Published:** 2020-06-23

**Authors:** Carole Laffont, Ariel Ivanovici, Pierre Gautrat, Mathias Brault, Michael Anthony Djordjevic, Florian Frugier

**Affiliations:** 1 Institute of Plant Sciences Paris-Saclay (IPS2), CNRS, INRA, Univ d’Evry, Université de Paris; Université Paris-Saclay, Gif-sur-Yvette, France; 2 Division of Plant Sciences, Research School of Biology, The Australian National University, Canberra, ACT 2601 Australia

**Keywords:** Plant sciences, Rhizobial symbiosis

## Abstract

Legumes tightly regulate nodule number to balance the cost of supporting symbiotic rhizobia with the benefits of nitrogen fixation. C-terminally Encoded Peptides (CEPs) and CLAVATA3-like (CLE) peptides positively and negatively regulate nodulation, respectively, through independent systemic pathways, but how these regulations are coordinated remains unknown. Here, we show that rhizobia, Nod Factors, and cytokinins induce a symbiosis-specific *CEP* gene, *MtCEP7*, which positively regulates rhizobial infection. Via grafting and split root studies, we reveal that MtCEP7 increases nodule number systemically through the MtCRA2 receptor. *MtCEP7* and *MtCLE13* expression in rhizobia-inoculated roots rely on the MtCRE1 cytokinin receptor and on the MtNIN transcription factor. MtNIN binds and transactivates *MtCEP7* and *MtCLE13*, and a NIN Binding Site (NBS) identified within the proximal *MtCEP7* promoter is required for its symbiotic activation. Overall, these results demonstrate that a cytokinin-MtCRE1-MtNIN regulatory module coordinates the expression of two antagonistic, symbiosis-related, peptide hormones from different families to fine-tune nodule number.

## Abstract (web summary)

CLE and CEP peptides regulate rhizobial symbiosis in legumes to balance the benefits of nitrogen fixation with the metabolic costs of nodule production. Here Laffont et al. show that cytokinin and bacterial Nod factors induce *Medicago* CEP7 which acts antagonistically to CLE13 to fine-tune nodulation.

## Introduction

About 60 million years ago, members of the *Fabaceae* (legume) plant family evolved the ability to enter into a symbiotic relationship with nitrogen-fixing soil bacteria, generically called rhizobia, to build a dedicated organ on their root system, the root nodule^1^. When mineral nitrogen is limiting in soils, symbiotic rhizobia can provide the nitrogen necessary to support plant growth from the unlimited atmospheric reservoir, thus giving legume plants a competitive advantage in these environments. Legume root nodules initiate following the secretion of rhizobial Nod factor (NF) signals that are perceived at the root epidermis, preferentially in a susceptible region located above the root apical meristem in compatible host plants^2–4^. This symbiotic partner recognition triggers a signaling cascade that activates the rhizobial infection of root hairs and subsequently the formation of infection threads (ITs) that grow toward root inner cortical cells. Simultaneously, rhizobium and NF perception (NFP) at the root epidermis activate cell divisions mostly in the inner cortical and pericycle cells to initiate a nodule organ primordium^5,6^, which is reached by growing ITs filled with rhizobia. This primordium then differentiates into a root nodule to accommodate the nitrogen-fixing rhizobia. The legume plant then provides carbon sources to rhizobia, as well as a low oxygen environment that is required to enable the bacterial nitrogenase to fix atmospheric nitrogen in root nodules.

Rhizobial NF signaling in host plant roots rapidly activates the transcription of early nodulation genes such as *ENOD11* (for *early nodulin 11*) in the root epidermis. *ENOD11* expression then follows IT progression from the root epidermis to the cortex^7^. Therefore, *ENOD11* can be considered as a marker for early symbiotic signaling activation and rhizobial infections^8^. Downstream of NF signaling activation, nodule organogenesis requires cytokinin since cytokinin receptor loss-of-function mutants (e.g., *lotus histidine kinase 1* [*lhk1* in *Lotus japonicus*] or *cytokinin response 1* [*cre1* in *Medicago truncatula*]) have a reduced nodulation capacity^9–13^. Consistently, the constitutive activation of LHK1 in the *L. japonicus spontaneous nodule formation2* (*snf2*) mutant triggers ectopic cortical cell divisions and spontaneous nodulation in the absence of rhizobia^14^. Cytokinin additionally negatively regulates rhizobial infections or NF signaling in *L. japonicus* and *M. truncatula*, respectively^10,15–17^.

Combined molecular and genetic analyses allowed the identification of genes critical for nodule initiation. Among those, a *L. japonicus* mutant defective in rhizobial entry into root hairs was affected in the nodule inception (NIN) transcription factor (TF)^18^. *NIN* expression is rapidly activated after the perception of NF, and a root hair-specific transcriptomic analysis suggests that NIN regulates rhizobial infections^18–20^. Accordingly, NIN is required for the initiation of symbiotic infections in root hairs^21^. In addition, *NIN* expression is induced rapidly by cytokinin depending on the CRE1 receptor^9,11^, and the constitutive expression of *NIN* in *L. japonicus* is sufficient to activate ectopic cortical cell divisions in the absence of rhizobia^22^. Furthermore, *M. truncatula* NIN also restricts the extent of *ENOD11* expression in the root rhizobial susceptible zone, and *nin* presumptive null mutants are impaired in IT formation^18,23^. The *L. japonicus nin*^*daphne*^ allele, however, shows a non-nodulation phenotype associated with a high number of ITs, indicating that the root zone susceptible for rhizobial infection is enlarged in *daphne*^24^. The *daphne* nodulation phenotype is associated with a broader epidermal expression of *NIN* than in the wild type (WT). In *M. truncatula*, a *daphne-like* mutant was recently identified, and the NIN coding sequence expressed from a 5 kb proximal *NIN* promoter region was sufficient to rescue its hyperinfection phenotype, even though this region was not sufficient to restore nodule organogenesis^25^. Indeed, an additional remote *cis*-regulatory sequence located 18 kb upstream from the *NIN* gene start codon was required to complement nodule organogenesis of the *daphne-like nin* mutant. Hence, NIN coordinates NF responses, rhizobial infection, and nodule organogenesis.

NIN additionally controls nodule number, since this TF triggers the expression of secreted Clavata3/embryo surrounding region (CLE) signaling peptides involved in the negative autoregulation of nodulation (AON) pathway^26^. These AON signaling peptides are referred to as CLE12 and CLE13 in *M. truncatula*, CLE-RS (CLE-root signal) 1, CLE-RS2, and CLE-RS3 in *L. japonicus*, and RIC (rhizobium-induced CLE) in *Glycine max*^27–31^. *CLE-RS1* and *CLE-RS2* genes are expressed within 1 day post rhizobial inoculation^32^, and both in *L. japonicus* and *G. max*, NIN directly binds promoters and activates the expression of these nodulation-related CLE peptide-encoding genes to initiate the long distance (systemic) AON pathway^31,32^. Shoot-to-root AON signals then generate a negative feedback loop on *NIN* expression, thus allowing a homeostatic regulation of the root nodule number^32^. CLE peptides produced in roots inoculated with rhizobia are proposed to be translocated through the xylem vasculature to the shoot where they are perceived by the leucine-rich repeat-receptor like kinase (LRR-RLK) SUNN (super numeric nodules) in *M. truncatula* or HAR1 (hypernodulation and aberrant root 1) in *L. japonicus*^33–36^. The activation of this systemic root-to-shoot pathway leads to the production of shoot-to-root signals that further inhibit the formation of new nodules in roots^2,16,37^.

Beside this negative systemic AON pathway, C-terminally encoded peptides (CEPs) regulate an independent systemic pathway in *M. truncatula* that promotes rhizobial infections and nodule number^3,38–41^. Low nitrogen and nitrogen starvation enhances the expression of several *Arabidopsis thaliana* and *M. truncatula CEP* genes, indicating that activation of the CEP pathway is tightly linked to nitrogen deficiency^37,38,42^. CEP peptide activation of nodulation depends on the LRR-RLK compact root architecture 2 (CRA2) acting in shoots^39^. Indeed, the exogenous application or overexpression of *M. truncatula* CEP1 peptides promote nodulation^3,38,40^, and *cra2* mutants form a reduced number of nodules^39,41^. The closest homolog of CRA2 in Arabidopsis, CEPR1 (CEP receptor 1), directly binds CEP peptides and mediates an N-demand systemic signaling pathway^43^ and the allocation of nutrients to roots^44^. In *M. truncatula*, a functional redundancy between CEP peptide-encoding genes required for the regulation of nodulation is anticipated since an RNA interference (RNAi) silencing of *CEP1* and *CEP2* genes did not reveal any nodulation phenotype, despite an increased lateral root phenotype was clearly observed, validating the efficient knockdown of these genes^38^. Symbiosis-specific *CEP* genes required for the positive control of root nodulation thus remain to be characterized.

In this study, we identify a *M. truncatula* CEP peptide-encoding gene, *MtCEP7*, with a unique symbiotic expression pattern. *MtCEP7* is induced rapidly in response to rhizobia or by short-term treatments with NF or cytokinin. *MtCEP7* expression depends on the MtCRE1 cytokinin receptor and on the MtNIN TF, and we show that MtNIN binds to the *MtCEP7* and *MtCLE13* promoters and can transactivate their expression. The addition of MtCEP7 peptides promotes nodulation systemically through the MtCRA2 receptor, and consistently, *MtCEP7* downregulation reduces nodule number and the number of rhizobial infections. *MtCEP7* expression is rapidly induced by rhizobia in the root epidermis and the 5 kb MtNIN promoter is sufficient to restore *MtCEP7* and *MtCLE13* induction by rhizobia, indicating that MtNIN activity induces the coordinated expression of specific nodulation-related signaling peptides from two different families. Strikingly, the expression of the *MtCEP7* gene driven by *MtCLE13* promoter sequences mitigates the onset of AON, suggesting that the balance between *MtCLE13* and *MtCEP7* expression might determine the number of successful infections in *M. truncatula* roots. Overall, these results indicate that a single cytokinin/MtCRE1/MtNIN regulatory module regulate two antagonistic, nodulation-related, signaling peptides, which allows a dynamic fine-tuning of the level of rhizobial infection and of the number of nodules.

## Results

### *MtCEP7* is induced by rhizobium, NFs, and cytokinin

**Fig. 1:**
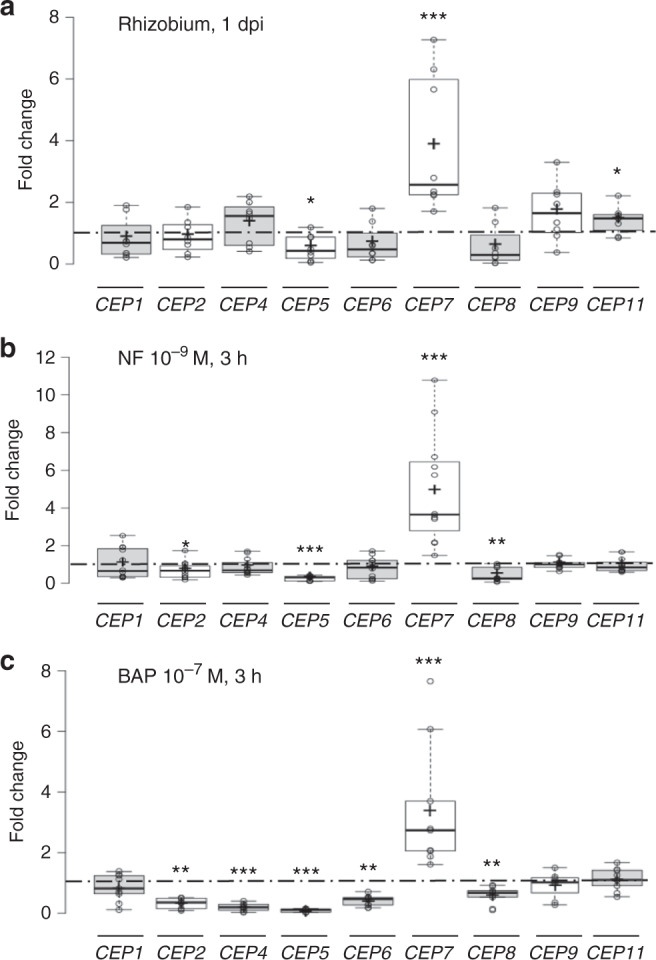
Expression of CEP peptide-encoding genes in response to rhizobia, Nod factors, or cytokinins. **a** Expression analysis of *CEP* genes in wild-type (WT) roots 1 day post rhizobial inoculation (1 dpi). qRT-PCR was used to measure the *CEP* gene expression levels, normalized relative to uninoculated roots. To highlight fold changes, the dotted line corresponds to a ratio of 1. Data points from four biological replicates are plotted as open circles (*n* = 8). **b** Expression analysis of *CEP* genes in WT roots in response to a Nod factor (NF) 10^−9^ M treatment for 3 h. qRT-PCR was used to measure the *CEP* expression levels in treated roots. The results were normalized relative to untreated roots. To highlight fold changes, the dotted line corresponds to a ratio of 1. Data points from six biological replicates are plotted as open circles (*n* ≥ 11). **c** Expression analysis of *CEP* genes in WT roots in response to a cytokinin (BAP 10^−7^ M) treatment for 3 h. qRT-PCR was used to measure the *CEP* gene expression levels in treated roots. The results were normalized relative to untreated roots. To highlight fold changes, the dotted line corresponds to a ratio of 1. Data points from four biological replicates are plotted as open circles (*n* ≥ 8). In **a**–**c**, center lines show the medians; box limits indicate the 25th and 75th percentiles as determined by the R software; whiskers extend 1.5 times the interquartile range from the 25th and 75th percentiles, outliers are represented by dots; and crosses represent sample means. Mann–Whitney test was used for each gene to assess significant differences between treated and control conditions, as indicated by asterisks (*α < 0.05; **α < 0.01; ***α < 0.001).

To identify CEP peptides involved in the regulation of symbiotic nodulation, we analyzed *CEP* gene expression in response to rhizobia as well as to NF or cytokinin signals that are required for nodule initiation. Only *MtCEP7* expression was induced within 1 day post inoculation (dpi) and in response to a short-term treatment with NFs (10^−9^ M, 3 h), whereas the other *CEP* genes were either weakly regulated or showed a reduced expression (e.g., *MtCEP5* and *MtCEP8*; Fig. 1a, b). An independent validation of the NF-dependent regulation of *MtCEP7* expression was obtained by using a mutant affecting the previously characterized *NFP* gene^45^. Indeed, the fast rhizobial induction of *MtCEP7* expression was abolished in the *nfp* mutant, as expected (Supplementary Fig. 1).

*CEP* gene expression was then analyzed in response to a 3-h treatment with another signal that promotes nodule initiation, namely, cytokinin (benzyl amino purine [BAP] 10^−7^ M). Once more, only *MtCEP7* was induced, whereas the expression of most of *CEP* genes (i.e., *MtCEP2*, *MtCEP4*, *MtCEP5*, *MtCEP6*, and *MtCEP8*) was reduced (Fig. 1c). These analyses therefore highlight that *MtCEP7* has a unique expression pattern, being rapidly induced by rhizobia as well as by the two key signals required for nodule initiation, NFs and cytokinin.

### *MtCEP7* symbiotic expression relies on MtCRE1 and MtNIN

**Fig. 2:**
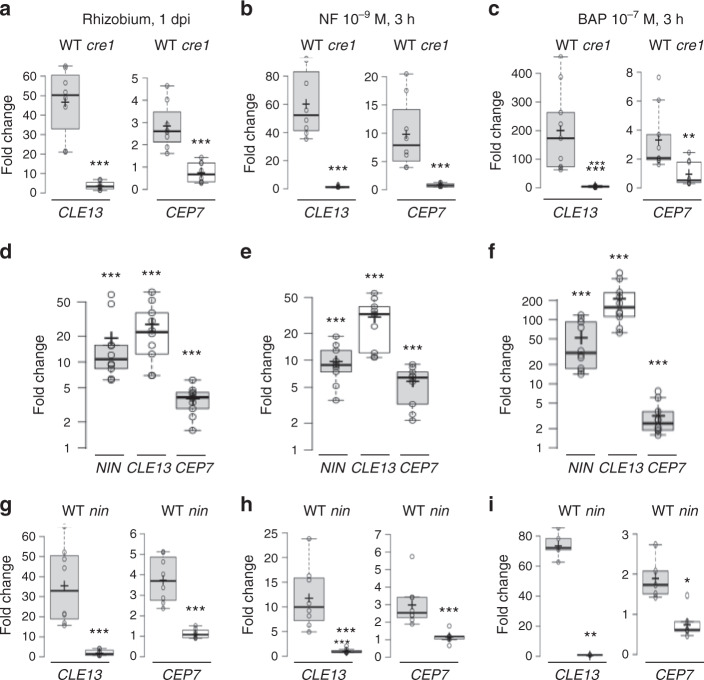
Rhizobia, Nod factor, and cytokinin regulation of *CEP7* and *CLE13* expression relies on the CRE1 cytokinin receptor and on the NIN transcription factor. **a**–**c** Expression analysis of the *MtCEP7* and *MtCLE13* genes in wild-type (WT) or *cre1* mutant roots in response to a 1 day post rhizobial inoculation (1 dpi) (**a**), to a Nod factor (NF) 10^−9^ M treatment for 3 h (**b**), or to a cytokinin (BAP 10^−7^ M) treatment for 3 h (**c**). qRT-PCR was used to measure the gene expression levels in treated roots, normalized relative to untreated roots. Data points from four biological replicates are plotted as open circles (*n* ≥ 8). **d**–**f** Expression analysis of *MtNIN*, *MtCLE13*, and *MtCEP7* genes in WT roots in response to a rhizobial inoculation 1 dpi (**d**), to an NF 10^−9^ M treatment for 3 h (**e**), or to a cytokinin (BAP 10^−7^ M) treatment for 3 h (**f**). qRT-PCR was used to measure the gene expression levels in treated roots, normalized relative to untreated roots. Data points from five biological replicates are plotted as open circles (*n* ≥ 10). **g**–**i** Expression analysis of *MtCLE13* and *MtCEP7* genes in WT and *nin* mutant roots in response to a rhizobial inoculation 1 dpi (**g**), to an NF 10^−9^ M treatment for 3 h (**h**), or to a cytokinin (BAP 10^−7^ M) treatment for 3 h (**i**). qRT-PCR was used to measure the gene expression levels in treated roots, normalized relative to untreated roots. Data points from at least three biological replicates are plotted as open circles (*n* = 8 except **i**, *n* ≥ 5). In **a**–**i**, center lines show the medians; box limits indicate the 25th and 75th percentiles as determined by the R software; whiskers extend 1.5 times the interquartile range from the 25th and 75th percentiles, outliers are represented by dots; and crosses represent sample means. Mann–Whitney test was used for each gene to assess significant differences between treatments in the WT and in the mutant (in **a**–**c** and **g**–**i**) or between treated and control conditions (in **d**–**f**), as indicated by asterisks (*α < 0.05; **α < 0.01; ***α < 0.001).

As the role of cytokinin in early nodulation relies predominantly on the MtCRE1 cytokinin receptor^11,12^, we investigated whether the rhizobia-, NF- and cytokinin-regulated *MtCEP7* expression depended on MtCRE1. The induction of *MtCEP7* by these treatments was strongly reduced in the *cre1* mutant (Fig. 2a–c). These results indicate that the rhizobia, NF, and cytokinin upregulation of *MtCEP7* expression depends on the MtCRE1 cytokinin receptor, which further links MtCEP7 to rhizobial infections. As the induction of *MtCLE13* in response to cytokinin depended on MtCRE1^46^, its expression was tested in response to a 24-h treatment with rhizobia and to a 3-h treatment with NFs or cytokinins. As expected, *MtCLE13* expression was induced by these treatments and was diminished in the *cre1* mutant (Fig. 2a–c).

As the expression of *MtNIN* was previously shown to be induced by both NF and cytokinin treatments^9,11,12^ and that *MtCLE13* expression depends on MtNIN^46^, we analyzed the ability of MtNIN to regulate *MtCLE13* and *MtCEP7* expression. The results showed that the upregulation of *MtNIN* expression by rhizobia, NF, or cytokinin treatments was consistent with the rapid induction of *MtCEP7* and *MtCLE13* expression (Fig. 2d–f), suggesting that these regulations might be mediated by MtNIN. By contrast, an analysis of *MtCEP7* and *MtCLE13* expression after a rhizobial, NF or cytokinin treatment in the *nin* mutant revealed that *MtCLE13* and *MtCEP7* gene induction was compromised (Fig. 2g–i). Overall, these results indicate that the MtCRE1 cytokinin receptor and the MtNIN TF are required for the regulation of *MtCLE13* and *MtCEP7* expression by rhizobia, NFs, and cytokinins.

### *MtCEP7* is induced in the root epidermis by rhizobium

**Fig. 3:**
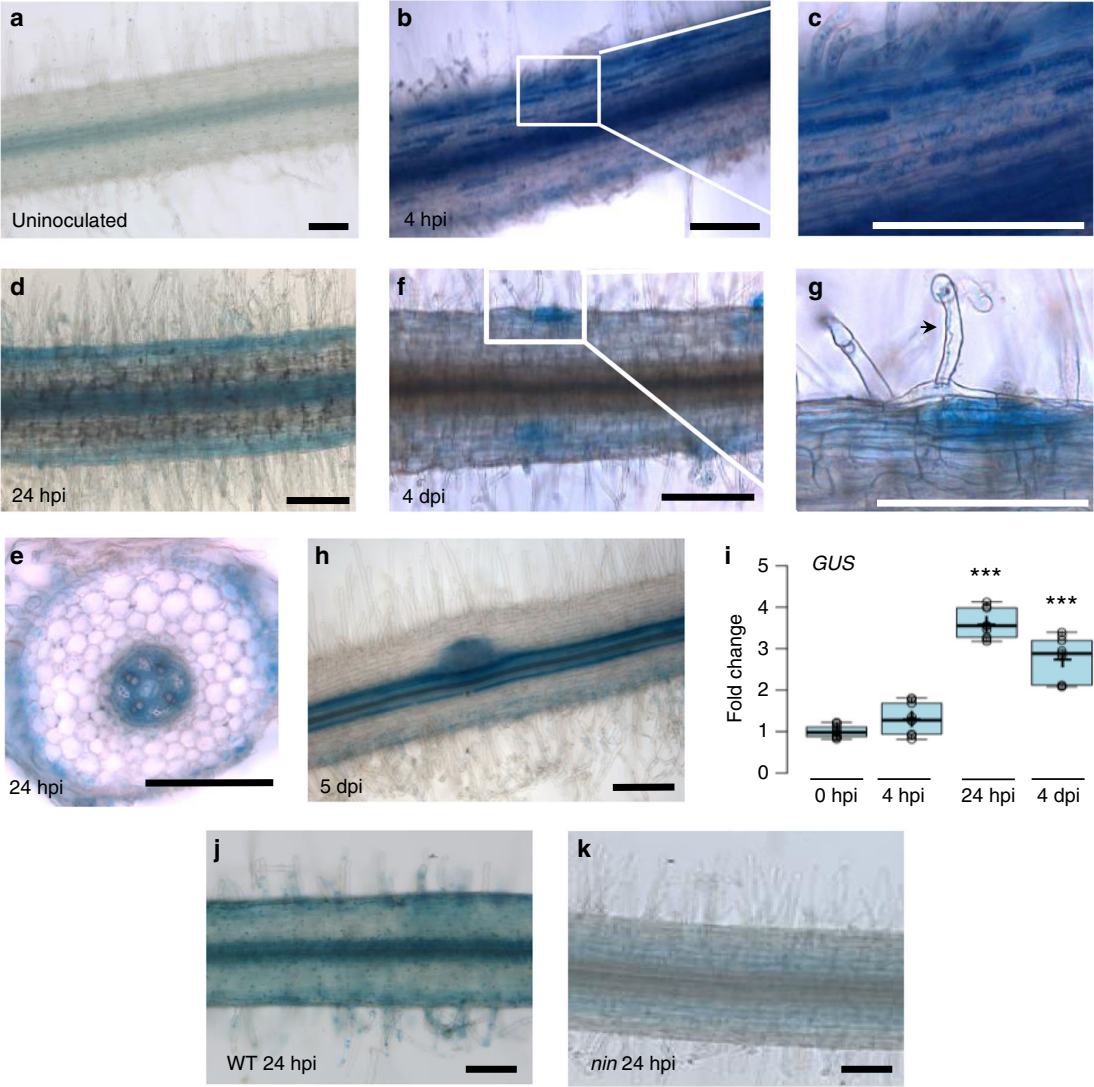
*MtCEP7* expression pattern in rhizobium-inoculated roots. **a**–**h** Expression pattern of a *pCEP7:GUS* transcriptional fusion. The GUS activity was analyzed in roots uninoculated (**a**) or inoculated with rhizobia 4 h post inoculation (hpi; **b**, **c**), 24 hpi (**d**, **e**), 4 days post rhizobial inoculation (dpi; **f**, **g**), or in a nodule primordium (5 dpi; **h**). **c** is a detail of **b**, and **g** is a detail of **f**, as indicated by white squares. All images show longitudinal roots, except e that is a transversal section. All images show the GUS staining as a blue signal in bright field microscopy. The arrowhead in **g** indicates an infection thread. At least eight independent roots were analyzed for each condition from five independent experiments. Scale bars = 200 µm. **i** Expression analysis of *GUS* transcripts in *pCEP7:GUS* transgenic roots, assessed by qRT-PCR. Expression levels were normalized relative to uninoculated roots (0 hpi). To highlight fold changes, the dotted line corresponds to a ratio of 1. Center lines show the medians; box limits indicate the 25th and 75th percentiles as determined by the R software; whiskers extend 1.5 times the interquartile range from the 25th and 75th percentiles, outliers are represented by dots; crosses represent sample means; and data points from three biological replicates are plotted as open circles (*n* = 9). Mann–Whitney test was used to assess significant differences between each time point and the non-treated control, as indicated by asterisks (***α < 0.001). **j**, **k** Expression pattern of the *pCEP7:GUS* transcriptional fusion in WT (**j**) versus *nin* mutant roots 4 dpi (**k**). In **a**–**h**, **j**, **k**, a minimum of eight independent roots per experiment were analyzed for each time point, and five independent experiments were performed. Images show the GUS staining as a blue signal in bright field microscopy. Scale bars = 200 µm.

*MtNIN* and *MtCLE13* genes were previously shown to be expressed in the epidermis as well as in the cortex of roots inoculated with rhizobia^15,25,28^, whereas the *MtCEP7* expression pattern was never investigated. We used a *pCEP7:GUS* transcriptional fusion to determine the spatiotemporal expression of *MtCEP7*. Uninoculated roots showed basal expression in the stele (Fig. 3a), whereas the rhizobial inoculation rapidly induced *pCEP7:GUS* expression in epidermal cells within 4–24 h post inoculation (hpi; Fig. 3b–e). At 4 dpi, *MtCEP7* expression then followed the progression of rhizobial infections and was detected in nodule primordia (Fig. 3f–h). In mature nodules, *MtCEP7* expression was expressed in dividing cells at the nodule apex and in the vascular bundles (Supplementary Fig. 2A, B). Concordantly, a quantitative reverse transcriptase polymerase chain reaction (qRT-PCR) analysis of the *GUS* transgene revealed the increased activity of the *MtCEP7* promoter in roots inoculated with rhizobia (24 hpi and 4 dpi; Fig. 3i). As anticipated from previous qRT-PCR analyses, the symbiotic expression of *MtCEP7* in the root epidermis is detected in the WT but not in the *nin* mutant (Fig. 3j, k).

### MtNIN binds and transactivates *MtCLE13* and *MtCEP7*

**Fig. 4:**
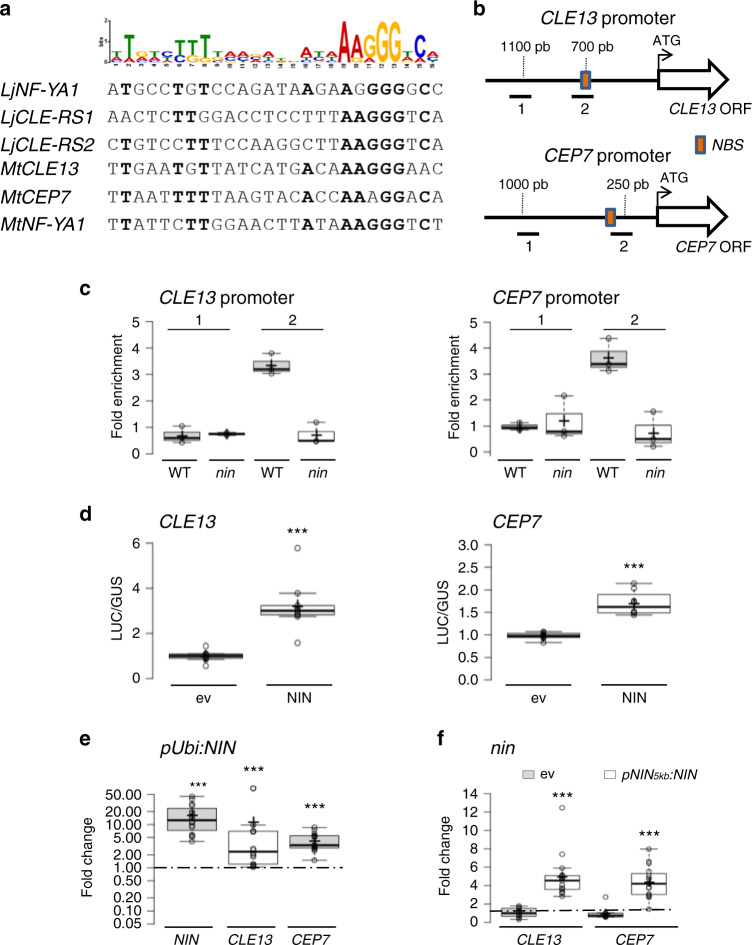
NIN binds promoters and transactivates the expression of *CLE13* and *CEP7*. **a** Alignment of NIN-binding site (NBS) motifs previously identified in *LjNF-YA1*, *LjCLE-RS1*, and *LjCLE-RS2* promoters^32^ with homologous regions of *MtNF-YA1*, *MtCLE13*, and *MtCEP7* promoters. Bold characters represent nucleotides conserved in more than four sequences out of the six identified. The sequence logo is derived from the six candidate NBSs, aligned using the MEME algorithm (http://meme-suite.org/index.html). **b** Schematic representation of *MtCLE13* and *MtCEP7* genes highlighting promoter regions used for ChIP-qPCR analyses. Red boxes represent the predicted NBSs identified on each promoter sequence. **c** NIN binds to the *MtCLE13* and *MtCEP7* promoters. Chromatin immunoprecipitation (ChIP)-qPCR analysis of NIN binding to *MtCLE13* (left panel) and *MtCEP7* (right panel) promoters, in wild-type (WT) or *nin* mutant roots 5 days post rhizobium inoculation (dpi) using either an anti-NIN antibody or IgG as a negative control. The fold enrichment of NIN binding was determined relative to IgG (control) IPs. One representative biological replicate out of two is shown. Data points from three technical replicates are plotted as open circles. **d** NIN transactivates *MtCLE13* and *MtCEP7* gene expression in *M. truncatula* mesophyll protoplasts. The promoter regions of *MtCLE13* (left panel) and *MtCEP7* (right panel) were fused to the luciferase (LUC) reporter gene and co-transformed in protoplasts with a p35S:NIN or an empty vector (ev). An AtUbi:GUS construct was used to measure the transformation efficiency. LUC/GUS ratios were normalized relative to values obtained in protoplasts transformed with an ev. To highlight fold changes, the dotted line corresponds to a ratio of 1. Data points from three biological replicates are plotted as open circles (*n* = 6). Mann–Whitney test was used for each promoter to assess significant differences between LUC/GUS ratios in the presence or absence of the NIN construct, as indicated by asterisks (***α < 0.001). **e** NIN activates *MtCLE13* and *MtCEP7* gene expression in *M. truncatula* roots. Expression analysis by qRT-PCR of *MtNIN*, *MtCLE13*, and *MtCEP7* in wild-type (WT) uninoculated roots transformed with a pAtUbi:NIN construct or an ev. Plants were grown on a nitrogen-free Fåhraeus medium. To highlight fold changes, expression levels were normalized relative to roots transformed with the ev. Data points from six biological replicates are plotted as open circles (*n* ≥ 12). Mann–Whitney test was used for each gene to assess significant differences between pUbi:NIN-transformed roots and control roots, as indicated by asterisks (***α < 0.001). **f** *NIN* expression driven by the *pNIN*_*5kb*_ promoter is sufficient to restore the regulation of *MtCLE13* and *MtCEP7* gene expression in response to rhizobium. *nin* mutant roots were transformed with either an ev or the *pNIN*_*5kb*_*:NIN* construct. *MtCLE13* and *MtCEP7* gene expression was analyzed by qRT-PCR in roots grown in vitro on a nitrogen-free Fåhraeus medium, 2 dpi with rhizobium. Expression levels were normalized relative to uninoculated roots for each genotype. To highlight fold changes, the dotted line corresponds to a ratio of 1. Data points from five biological replicates are plotted as open circles (*n* ≥ 9). Mann–Whitney test was used for each gene to assess significant differences between roots transformed with the *pNIN*_*5kb*_*:NIN* construct and the control, as indicated by asterisks (***α < 0.001). In **c**–**f**, center lines show the medians; box limits indicate the 25th and 75th percentiles as determined by the R software; whiskers extend 1.5 times the interquartile range from the 25th and 75th percentiles, outliers are represented by dots; and crosses represent sample means.

As NIN directly regulates *CLE-RS* genes in *L. japonicus*^32^, we tested whether such regulation was conserved in *M. truncatula*, and whether it could be shared for the regulation of the *MtCEP7* gene. The analysis of *MtCLE13* and *MtCEP7* proximal promoters revealed the presence of a sequence similar to the NIN-binding site (NBS) previously described in *L. japonicus* (ref. ^32^; Fig. 4a, b). We thus performed chromatin immunoprecipitation (ChIP) in rhizobia-inoculated roots of WT plants and *nin* mutants, with a native antibody targeting MtNIN previously validated in Vernié et al.^47^, or with immunoglobulin G (IgG) used as a negative control to evaluate non-specific binding. The IP enrichment of each promoter was measured by quantitative PCR (qPCR) using primers designed in the proximal regions of the *MtCLE13* and *MtCEP7* promoters that contained the predicted NBS *cis*-element identified, or in distal regions (Fig. 4b). An enrichment of the proximal region of the *MtCLE13* promoter was detected in the MtNIN IP, compared to the more distal region (Fig. 4c, left panel). This enrichment was not detected for MtNIN IPs performed in the *nin* mutant, as expected for a negative control. This result indicates a conservation of the NIN interaction with the promoter of a nodulation-related CLE peptide encoding gene between *M. truncatula* and *L. japonicus*. Furthermore, the MtNIN ChIP also revealed enrichment for the *MtCEP7* proximal promoter region, relative either to the more distal promoter region or to the MtNIN IP performed in the *nin* mutant, as negative controls (Fig. 4c, right panel). This suggests that NIN co-regulates *MtCLE13* and *MtCEP7* expression. In order to determine whether MtNIN is sufficient to transactivate *MtCLE13* and *MtCEP7* gene expression, transient transformation assays were performed in *M. truncatula* mesophyll protoplasts (Fig. 4d). To this aim, either a *p35S:3xHA-NIN* construct or the corresponding empty vector was co-transformed with a luciferase reporter gene driven either by the *MtCLE13* or the *MtCEP7* promoter region. After normalization of transfection efficiency with a *pAtUbi:GUS* control, the *pMtCLE13:LUC* and *pMtCEP7:LUC* activities were found as significantly enhanced by MtNIN ectopic expression (Fig. 4d). These results indicate that MtNIN transactivates the expression of *MtCLE13* and *MtCEP7*.

**Fig. 5:**
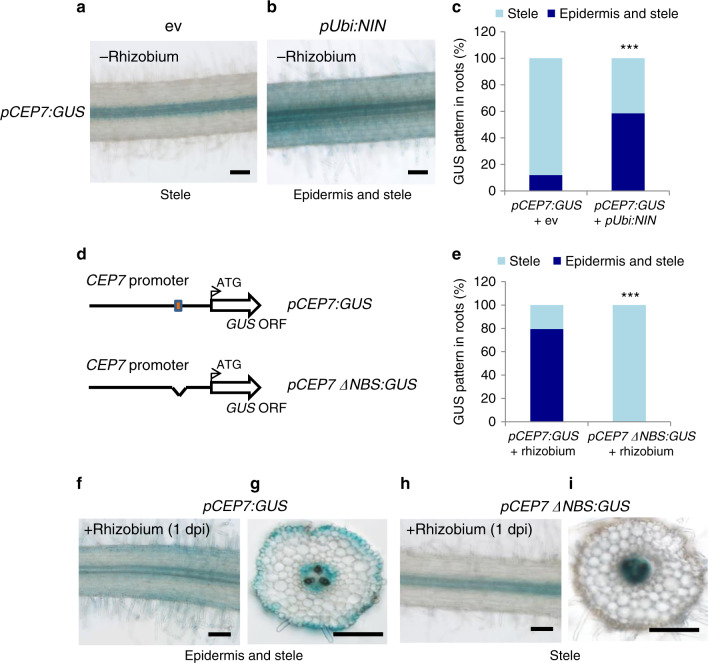
The NIN-binding site *cis*-element predicted in the proximal region of the *CEP7* promoter is required for its induction by rhizobia. **a**–**c** *NIN* expression driven by the *pUbi* promoter is sufficient to induce the expression of a *MtCEP7:GUS* fusion in the absence of rhizobium (**b**), compared to an empty vector (ev) control (**a**). Representative images of the *GUS* expression pattern associated only with the root stele (“stele”) or with both the root epidermis and stele (“epidermis and stele”). Wild-type plants were grown in vitro on a nitrogen-free Fåhraeus medium. In c, quantification of the proportion of roots (*n* ≥ 25) showing a *GUS* expression pattern associated only with the root stele or with both the root epidermis and stele, as described in **a**, **b**. **d**–**h** The NIN-binding site (NBS) in the proximal region of the *MtCEP7* promoter is required for the induction of a *MtCEP7:GUS* fusion in response to rhizobium. In **d**, schematic representation of the *MtCEP7* promoter, highlighting the predicted NBS *cis*-element identified as a red box and the corresponding deletion construct (*pCEP7ΔNBS:GUS*). In **e**, quantification of the proportion of roots (*n* ≥ 34) showing a *GUS* expression pattern associated only with the root stele (“stele”) or with both the root epidermis and stele (“epidermis and stele”), as described in **f**–**i**. In **f**, **g**, representative images of the *GUS* expression pattern associated only with the root stele or with both the root epidermis and stele of *pCEP7ΔNBS:GUS* 1 day post rhizobium inoculation (dpi) in longitudinal (**f**) or transversal (**g**) sections. In **h**, **i**, representative images for the same *GUS* expression patterns for *MtCEP7:GUS* roots 1 dpi in longitudinal (**h**) or transversal (**i**) sections. In **a**, **b**, **f**–**i**, a minimum of eight independent roots were analyzed for each time point from three independent experiments. Images show the GUS staining as a blue signal in bright field microscopy. Scale bars = 100 µm. In **c**, **e**, Fisher exact test was performed to assess significant differences in the proportion of roots (*n* ≥ 25) showing the different *GUS* expression patterns between the NIN overexpressing (**c**), or NBS-deleted *MtCEP7:GUS* (**e**) roots, and the control, as indicated by asterisks (***α < 0.001).

To determine whether the ectopic expression of MtNIN could activate *MtCEP7* and/or *MtCLE13* expression in *M. truncatula* roots, *MtNIN* was constitutively expressed using the *AtUbi* promoter, and *MtNIN*, *MtCLE13*, and *MtCEP7* transcript levels were analyzed in uninoculated transformed roots (Fig. 4e). In these conditions, the high expression level of *MtNIN* corresponds to the transgene ectopic expression, which was sufficient to increase the expression of both *MtCLE13* and *MtCEP7* genes. Accordingly, ectopic expression of *MtNIN* from the *pUbi* promoter was sufficient to induce the expression of the *MtCEP7:GUS* fusion in the absence of any symbiotic treatment, notably in the root epidermis (Fig. 5a–c). The ectopic expression of *MtNIN* therefore bypasses the need for rhizobium, NF, or cytokinin treatments to induce *MtCLE13* and *MtCEP7* expression.

We next determined whether the MtNIN-dependent regulation of *MtCEP7* relied on the putative NBS *cis*-element previously identified in the proximal region of the promoter. To this aim, we generated a version of the *CEP7* promoter deleted from this NBS sequence and fused to the *GUS* reporter gene (Fig. 5d). After a short-term rhizobial inoculation (1 dpi), the symbiotic induction of the *pCEP7ΔNBS:GUS* promoter was abolished (Fig. 5e, h, i) compared to the WT promoter (Fig. 5e–g), notably in the root epidermis. This indicates that the NBS *cis*-element identified is required for the symbiotic regulation of *MtCEP7* expression.

Finally, to establish a link between *MtNIN* epidermal expression and the symbiotic regulation of *MtCEP7*, we used the recently characterized 5 kb *NIN* promoter region (*pNIN*_*5kb*_*:NIN*) that is expressed in the root epidermis but not in the root cortex and that can specifically rescue the infection phenotype of the *nin* mutant^25^. This *pNIN*_*5kb*_*:NIN* construct introduced in *nin* mutant roots was sufficient to restore expression of *MtCEP7* as well as of *MtCLE13* (Fig. 4f). These results suggest that the *MtNIN* expression domain associated with the root epidermis is sufficient to induce both *MtCEP7* and *MtCLE13* expression in response to rhizobia.

### MtCEP7 peptides promote nodulation and rhizobial infections

**Fig. 6:**
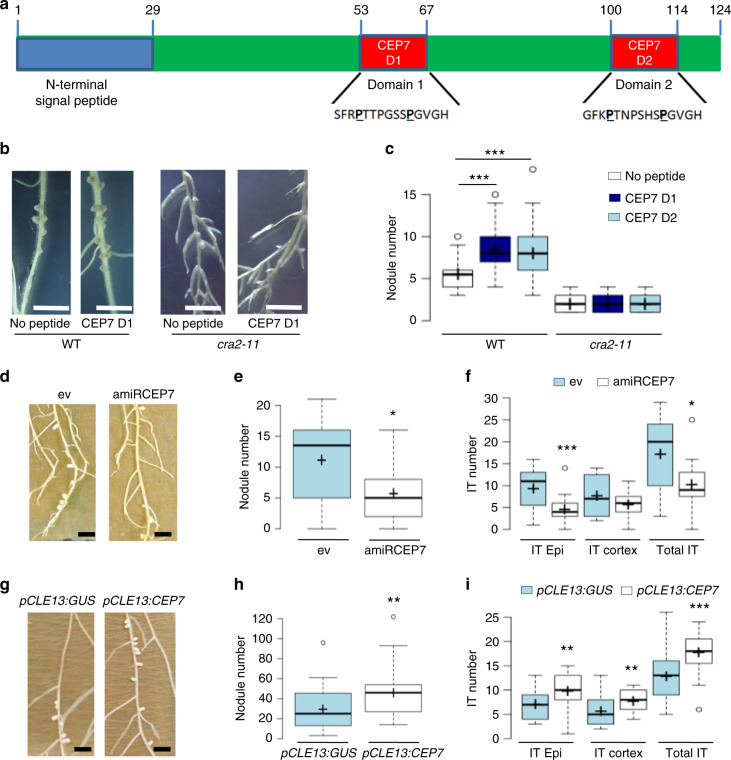
CEP7 peptides regulate positively nodule number and rhizobial infections. **a** Schematic representation of the predicted MtCEP7 prepropeptide. The blue rectangle represents the N-terminal signal peptide predicted using SignalP 3.0 (http://www.cbs.dtu.dk/services/SignalP-3.0/). Red rectangles represent the predicted MtCEP7 Domain 1 (CEP7 D1) and Domain 2 (CEP7 D2) peptide sequences with hydroxyl modifications to proline as indicated in bold. **b** Representative images of the nodulation phenotype of wild-type (WT) and *cra2* mutants treated with synthetic CEP7 D1 or CEP7 D2. Scale bars = 1 cm. **c** Quantification of the nodule number in WT and *cra2* mutants, grown in vitro on a nitrogen-free Fåhraeus medium supplemented or not with 1 µM CEP7 D1 or CEP7 D2 peptides. Nodule number was scored at 14 days post rhizobium inoculation (dpi). Graphs are based on data points from three biological replicates (*n* ≥ 59). Mann–Whitney test was used for each genotype to assess significant differences between peptide-treated and control plants, as indicated by asterisks (***α < 0.001). **d** The nodulation phenotype of plants expressing an artificial microRNA targeting *MtCEP7* transcripts (amiRCEP7). Representative images of nodulated roots transformed either with an empty vector (ev) or with the amiRCEP7 construct described in **d**. Scale bars = 1 cm. **e** Quantification of the nodule number of plants transformed either with an ev or with an amiRCEP7 construct. Plants were grown in vitro on a nitrogen-free Fåhraeus medium and nodules were scored at 14 dpi. Graphs are based on data points from 1 representative biological replicate out of 3 (*n* ≥ 14). Mann–Whitney test was used to assess significant differences, as indicated by asterisks (*α < 0.05). **f** Quantification of infection thread (IT) number in six dpi roots transformed either with an ev or with the amiRCEP7 construct and inoculated with a rhizobium strain constitutively expressing a LacZ reporter. ITs that reached the epidermis (epidermal ITs) were distinguished from ITs that reached the cortex (cortical ITs). Graphs are based on data points from two biological replicates (*n* ≥ 15). Mann–Whitney test was used to assess significant differences between amiRCEP7 roots and control roots, as indicated by asterisks (*α < 0.05; ***α < 0.001). **g** Representative images of the nodulation phenotype of plants transformed either with a *pCLE13:GUS* or a *pCLE13:CEP7* construct. Scale bars = 1 cm. **h** Quantification of the nodule number in plants transformed either with the *pCLE13:CEP7* construct or the *pCLE13:GUS* construct as a negative control. Plants were grown in pots containing a perlite/sand (3/1) mixture and watered with a “i” low nitrogen medium, and nodules were scored at 21 dpi. Graphs are based on data points from two biological replicates (*n* ≥ 28). Mann–Whitney test was used to assess significant differences, as indicated by asterisks (**α < 0.01). **i** Quantification of IT number in six dpi roots transformed either with the *pCLE13:CEP7* construct or the *pCLE13:GUS* construct as a negative control and inoculated with a rhizobium strain constitutively expressing a LacZ reporter. ITs that reached the epidermis (epidermal ITs) were distinguished from ITs that reached the cortex (cortical ITs). Graphs are based on data points from two biological replicates (*n* ≥ 15). Mann–Whitney test was used to assess significant differences between the *pCLE13:CEP7* roots and control roots, as indicated by asterisks (**α < 0.01; ***α < 0.001). In **c**, **e**, **f**, **h**, **i**, center lines show the medians; box limits indicate the 25th and 75th percentiles as determined by the R software; whiskers extend 1.5 times the interquartile range from the 25th and 75th percentiles, outliers are represented by dots; and crosses represent sample means.

To evaluate the function of MtCEP7 peptides in early nodulation, exogenous treatments were first performed using peptide sequences corresponding to the conserved Domain 1 (MtCEP7 D1) or Domain 2 (MtCEP7 D2) regions (Fig. 6a, Supplementary Fig. 3A). A similar enhancement of the number of nodules was observed compared to non-treated roots (Fig. 6b, c). As effects on nodulation of another CEP peptide, MtCEP1, were previously shown to rely on the MtCRA2 CEP receptor homolog^39,41^, MtCEP7 peptides were thus exogenously applied on *cra2* mutant roots. Positive effects on nodule number induced both by MtCEP7 D1 and D2 peptides relied on the MtCRA2 pathway (Fig. 6b, c). Grafting experiments revealed that the activity of the MtCRA2 receptor was required in shoots to mediate the MtCEP7 positive effect on nodule number (Supplementary Fig. 4A). In addition, split-root experiments were conducted where only half of the root system was treated with exogenous MtCEP7 peptides but nodule numbers quantified in parallel in locally treated roots and in distant non-treated (systemic) roots. This analysis revealed that the number of nodules was increased not only in MtCEP7-treated roots but also systemically in non-treated roots (Supplementary Fig. 4B). Collectively, these results indicate that MtCEP7 regulates positively nodule number depending on the MtCRA2 receptor and that this regulation occurs systemically in the root system thanks to the action of MtCRA2 in shoots.

To independently validate the function of MtCEP7 in early nodulation, loss-of-function approaches were additionally performed. Two independent strategies were used, namely, the silencing of *MtCEP7* expression by an RNAi (*MtCEP7* RNAi) or by an artificial microRNA (amiRNA) approach (amiRCEP7; regions targeted by each construct are shown in Supplementary Fig. 3B). The number of nodules of *MtCEP7* RNAi roots was lower than for *GUS* RNAi control roots (Supplementary Fig. 5A), which agrees with results of the previous exogenous peptide treatments. Similarly, amiRCEP7 roots formed a reduced number of nodules compared to control roots (Fig. 6d, e). The strength of nodule number phenotypes was correlated to the efficiency and specificity of these approaches, evaluated by qRT-PCR on the different CEP peptide-encoding genes (Supplementary Fig. 5B, C). Collectively, these results suggest that the MtCEP7 peptide has a positive role in nodulation but that a redundant function with other CEP peptide-encoding genes likely exists.

Since MtNIN expression in the root epidermis restores the symbiotic induction of *MtCEP7* expression (Fig. 4f), the role of MtCEP7 peptides in the regulation of rhizobial infections was further investigated using the amiRCEP7 construct shown previously to affect more strongly nodule number. To this aim, we used a *Sinorhizobium* strain expressing constitutively a LacZ marker to visualize infection events in roots (Fig. 6f). The number of ITs was reduced in roots expressing the amiRCEP7 construct relative to control roots, mostly in the root epidermis. As an independent validation, a stable line expressing the *pENOD11-GUS* symbiotic infection marker was used (Supplementary Fig. 6). The number of *MtENOD11*-positive infection foci was similarly significantly reduced in amiRCEP7 roots, mostly in epidermal cells but also in cortical cells (Supplementary Fig. 6A, B). Collectively, these results indicate that the MtNIN-dependent induction of *MtCEP7* expression positively regulates rhizobial infections.

To evaluate the functional relevance of the symbiotic activation of MtCEP7, we used the *MtCLE13* promoter to ectopically express *MtCEP7* in the same spatiotemporal symbiotic domain as this negatively acting CLE peptide^28,36^. The *MtCEP7* expression level increased ~35-fold in *pCLE13*:*MtCEP7* roots compared to p*CLE13:GUS* control roots (Supplementary Fig. 5D) and was associated with a significant increase in the number of nodules formed on p*CLE13:CEP7* roots relative to control roots (Fig. 6g, h). In addition, the number of ITs was evaluated, using the LacZ-expressing *Sinorhizobium* strain (Fig. 6i), revealing a significant increase in p*CLE13:CEP7* roots relative to control roots both in the epidermis and in the cortex. Overall, these results suggest that increasing the level of MtCEP7 activity may mitigate the CLE13-dependent negative AON regulation of nodule number and symbiotic infections.

## Discussion

In this study, we show that the *MtCEP7* gene is rapidly upregulated by rhizobia as well as by NFs and cytokinins, suggesting that *MtCEP7* may have a function more specifically related to symbiotic nodulation. A combination of gain-of-function experiments, based on exogenous treatment applications and ectopic expression, as well as of loss-of-function experiments, based on RNAi and amiRNA strategies, support a positive function of MtCEP7 in promoting nodulation and rhizobial infections. The previously observed increased nodulation induced by the ectopic expression of *MtCEP1* or by the addition of MtCEP1 peptides mimics the effect of MtCEP7^3,38,40^, even though a *MtCEP1* knock-down affecting also *MtCEP2* expression does not alter nodulation, despite promoting lateral root formation and thus indicating the efficiency of the RNAi approach^38^. Among all other CEP-encoding genes, the unique feature of the *MtCEP7* gene to be induced by rhizobia supports a role in maintaining a window for new nodule formation after the initial rhizobial infections occurred. Given that legume symbiotic nodulation emerged during evolution after the emergence of *CEP*^48^ and *CLE* genes^49^, the identification of a NF-dependent symbiotic expression pattern for *MtCEP7*, or *MtCLE12* and *MtCLE13*, suggests a sub-functionalization of these genes within their respective families. The loss-of-function approaches, however, highlight the possibility that a functional redundancy of *MtCEP7* likely exist with other CEP encoding gene(s) that is (are) not upregulated rapidly after *Sinorhizobium meliloti* inoculation.

The fast induction of *MtCEP7* expression in response to rhizobia led us to analyze its regulation in response to NF signals. In agreement with RNAseq datasets from Jardinaud et al.^15^ showing an NF induction of *MtCEP7* in the root epidermis, we now show that rhizobia also rapidly induces the expression of this gene in a subset of root epidermal cells. This expression then follows the progression of rhizobial infections to finally reach the growing nodule primordium. Since the MtCRA2 and MtSUNN receptors are not expressed in the root epidermis either with or without NF^15^, the symbiotically induced expression of *MtCEP7* and *MtCLE13* likely acts non-cell autonomously. Previous work indicated that MtCEP1 phenotypes relied on the activity in shoots of the CRA2 receptor^39,41^, which appears functionally orthologous to the CEPR1 CEP receptor of Arabidopsis^3,43,50^. Similarly, we now show using both hypocotyl grafts between the WT and the *cra2* mutant and split-root experiments with CEP7 peptides that the promotion of nodulation induced by MtCEP7 peptides relies on MtCRA2 and that this regulation occurs systemically in distant non-treated roots through the activity of the MtCRA2 receptor in shoots.

There is scarce knowledge on how peptide hormone signaling intersects with metabolite hormones, such as cytokinin^51,52^. An example in Arabidopsis is the regulation by WUSCHEL of *AtCLV3* expression in the shoot apical meristem, which is itself a direct target of cytokinin signaling^52,53^. The *M. truncatula* model offers a unique opportunity to dissect how two independent systemic signaling pathways regulate symbiotic nodulation antagonistically^36,41^. To our knowledge, we show here for the first time in plants that a single TF, MtNIN, which is upregulated by cytokinin, co-regulates the expression of two signaling peptides encoding genes from different families, *MtCEP7* and *MtCLE13*. Previously, MtCRE1 and MtNIN were shown to be required for the induction of *MtCLE13* in response to cytokinin^28,46^. We now show that MtCRE1 and MtNIN also are required for the induction of *MtCLE13* by NFs and rhizobia. This suggests that the direct regulation of AON-related *CLE* genes by NIN is evolutionary conserved between *L. japonicus* and *M. truncatula* (ref. ^32^; this study). Indeed, *MtCLE13* expression is transactivated by MtNIN in a leaf mesophyll protoplast assay, its promoter bound by MtNIN in a ChIP-qPCR assay, and the ectopic induction of MtNIN in *M. truncatula* roots is sufficient to induce *MtCLE13* expression. Strikingly, the same set of approaches revealed that MtNIN similarly binds the *MtCEP7* promoter and transactivates its expression. As reported in *L. japonicus*^32^, a conserved NBS *cis*-element was identified in the *MtCLE13* proximal promoter that corresponded to the NIN IP-enriched promoter region. Interestingly, an NBS *cis*-element was also identified in the NIN IP-enriched region of the *MtCEP7* proximal promoter, which is required for its short-term regulation by rhizobia in the root epidermis. Our results also suggest that MtNIN regulates the expression of *CLE13* and *CEP7* in the same expression domain notably including the root epidermis. The identification of this MtNIN-mediated co-regulation opens perspectives to extrapolate this type of co-regulation for other nutritional contexts and in non-legume plants.

It could be surprising that, at about the same time during early nodulation, MtNIN upregulates two signaling peptides that antagonistically regulate nodule number. It has to be noted, however, that the strength of the induction of *MtCEP7* expression by rhizobia is different than for MtCLE13^28^. This suggests that other TFs may be differentially recruited in addition to MtNIN to regulate *MtCLE13* versus *MtCEP7* expression. We used this differential symbiotic induction pattern to explore a potential functional relevance of this simultaneous induction of antagonistic nodulation-related signaling peptides by expressing *MtCEP7* with the *MtCLE13* promoter. Interestingly, an increased number of nodules was observed, suggesting that enhancing *MtCEP7* in the *MtCLE13* expression domain was able to mitigate the onset of the negative AON pathway. We thus speculate that the modulation of *MtCEP7* and *MtCLE13* expression through the rhizobium-/NF- and cytokinin-induced MtCRE1- and MtNIN-dependent signaling pathway may enable a dynamic fine-tuning of nodule number depending on plant’s metabolic capacities and needs. An attractive hypothesis is that the MtCEP7 pathway may be required to modulate the negative AON pathway during the plant life cycle, i.e., depending on growth needs that, for example, are driven by the shoot photosynthetic capacity. This implies that shoot-derived carbon signals may impact the MtCEP7 and MtCLE13 pathways, as recently shown in Arabidopsis for *CEP* genes^44^. In addition, an inhibitor of NIN function acting during early nodulation was identified recently in *G. max*, Nodule Number Control 1 (NNC1), which encodes an Apetala2-type TF that notably inhibits the NIN-dependent regulation of AON CLE peptide-encoding genes^31^. Further work is needed to determine whether this negative regulatory module is also recruited to modulate *MtCEP7* expression. In the future, we also need to better understand which molecular mechanisms allow environmental conditions or the plant metabolic status to impact the balance between CLE-dependent negative and CEP-dependent positive systemic pathways that fine tunes nodule number and the number of infection events.

## Methods

### Biological material, growth conditions, and treatments

The *M. truncatula* Jemalong A17 WT genotype, as well as the *cre1-1*, *nin-1*, *nfp-C31*, and *cra2-11* mutants were used in this study. These mutants are described in Plet et al.^11^, Marsh et al.^23^, Ben Amor et al.^45^, and Laffont et al.^41^, respectively. The *pENOD11:GUS* transgenic line was generated in Journet et al.^7^. Seeds were scarified in sulfuric acid (Sigma, www.sigmaaldrich.com) for 3 min, washed four times with distilled water, and then sterilized for 20 min in bleach (12% [v/v] sodium hypochlorite; Chlorifix, Bayrol, https://www.bayrol.fr/). After washings with sterile water, the seeds were placed on 1.5% water–agar plates (Bactoagar, Becton Dickinson), stratified for 2 days in the dark at 4 °C, and then germinated overnight at 24 °C. The germinated seedlings were grown in vitro vertically on square plates in a growth chamber at 24 °C under a long day photoperiod (16 h light; 150 μE intensity) on nitrogen-free Fåhraeus medium^54^. Alternatively, germinated seedlings were grown in pots containing a perlite:sand 3:1 mixture in a growth chamber (24 °C, 16 h light, 150 µmol m^−2^ s^−1^; relative humidity 60%) and watered every 2 days with the “i” low nitrogen medium (0.25 mM KNO_3_)^55^.

For nodulation experiments, the *S. meliloti* 1021 strain was used for in vitro experiments or the *Sinorhizobium medicae* WSM419 strain for plants grown in perlite/sand pots. To quantify infection events, a *S. meliloti* strain *Sm*1021 carrying a pXLGD4 plasmid expressing a *Pro*_*HemA*_*:LacZ* marker (GMI6526;^56^) was used to follow bacteria progression from root hairs to cortical cells. Five dpi roots were stained using a β-galactosidase histochemical assay as described in Ardourel et al.^56^ and observed using a stereomicroscope (Olympus, BX53, www.olympus-lifescience.com/).

In all cases, rhizobial strains were grown at 28 °C on a Yeast Extract Broth medium (YEB; Vervliet et al.^57^) supplemented with streptomycin 50 mg L^−1^ for *Sm*1021 or chloramphenicol 25 mg L^−1^ for WSM419, and plants were inoculated with an OD_600nm_ = 0.05 bacterial suspension. For the nodulation kinetic experiments, roots or developing nodules from at least eight individual plants per biological replicate were harvested 0, 1, 4, 8, and 14 dpi and frozen in liquid nitrogen for further RNA extraction.

For cytokinin and NF treatments, about 25 germinated seedlings were grown on a grid in a Magenta box filled with “i” low nitrogen liquid medium, under gentle agitation at 24 °C and with a long day photoperiod (24 °C, 16 h light, 150 µmol m^−2^ s^−1^). After 6 days, seedlings were treated or not with the cytokinin BAP 10^−7^ M (Sigma-Aldrich), or with NF 10^−9^ M, and grown under the same conditions. Treated or untreated roots (mock) from at least 10 individual plants per biological replicate were harvested after 3 h and immediately frozen in liquid nitrogen prior to RNA extraction.

For peptide treatments, about 25 germinated seedlings were grown on nitrogen-free Fåhraeus medium with or without 1 µM synthetic peptides corresponding to the conserved domains of MtCEP7 (GL Biochem Ltd, China): MtCEP7 Domain 1 (CEP7 D1), SFRhyPTTPGSShyPGVGH; and MtCEP7 Domain 2 (CEP7 D2), GFKhyPTNPSHShyPGVGH (where “hyP” indicates proline hydroxylation). Plants were grown in a growth chamber (22 °C, 16 h light, 100 µmol m^−2^ s^−1^).

Grafts were generated in vitro as described in the “Medicago handbook” (http://www.noble.org/medicagohandbook/) or according to the hypocotyl grafting technique described in Chapman et al.^50^ and then transferred after 2 weeks on nitrogen-free Fåhraeus medium with or without 1 µM synthetic MtCEP7 D1 peptides in the same growth conditions as previously described. Grafts were inoculated 1 week after transfer, and the nodule number was scored 7 and 14 days post *Sinorhizobium* inoculation.

Split roots were generated in vitro using Fåhraeus medium supplemented with 5 mM KNO_3_. Seedlings were grown for 24 h and root tips were cut and then grown for 4 days under the growth conditions previously described. Plants with two equivalent roots were then selected and transferred onto a nitrogen-free Fåhraeus medium where the agar was split. CEP7 D1 synthetic peptide (1 µM) was added, or not, to one half of the agar plate before the plants were transferred. Split roots were inoculated with rhizobia 3 days after transfer, and the nodule number was scored at 14 dpi in treated and untreated roots.

### Cloning and *Agrobacterium rhizogenes* root transformation

Using the Gateway technology, an RNAi construct targeting the *MtCEP7* gene was generated by PCR amplification of a 250-nt *MtCEP7* region (indicated by nucleotides in blue in Supplementary Fig. 2) using primers flanked with attB recombination sites (primers listed in Supplementary Table 1), which was inserted into the pDONR221 vector (ThermoFisher, www.thermofisher.com), subsequently recombined into the pFRN binary vector^9^, and then validated by sequencing. The pFRN GUS RNAi kanamycin-resistant vector previously described in Gonzalez-Rizzo et al.^9^ was used as a control. The efficiency of the MtCEP7 RNAi construct was evaluated on roots collected 2 days post rhizobia inoculation (dpi), immediately frozen in liquid nitrogen for further RNA extraction. For the *CEP7* promoter transcriptional fusion with *GUS*, a 2-kb region upstream of the predicted ATG was selected and amplified from genomic Jemalong A17 DNA, cloned into pENTR/D TOPO (ThermoFisher, http://www.thermofisher.com), sequenced, and subsequently transferred into the pKGWFS7 destination vector (spectinomycin/streptinomycin resistance for bacterial selection; ref. ^58^). The *p35S:3xHA-NIN* construct (carbenicillin resistant) was previously generated in Vernié et al.^47^, whereas the *pNIN*_*5kb*_*:NIN* construct and the corresponding empty vector (spectinomycin resistant) were generated in Liu et al.^25^.

Other constructs were generated using a Golden Gate strategy, as described in Engler and Marillonnet^59^, with vectors generated by Weber et al.^60^ and provided by the Engineering Nitrogen Symbiosis for Africa (ENSA) project (https://www.ensa.ac.uk/). For protoplast transformation, *pCEP7:LUC*, *pCLE13:LUC*, and *pAtUbi:GUS* single gene constructs (promoter–gene–terminator) were cloned into a level 1 Golden Gate vector (EC47811, ampicillin resistant). For *M. truncatula* root transformation*, pAtUbi:amiRCEP7*, *pCLE13:CEP7*, *pCLE13:GUS*, *pCEP7:GUS* and *pAtUbi:NIN:GFP* constructs were first cloned into the level 1 vector (EC47811), which was then combined with a *pNOS:Kanamycin* cassette (EC15029), to allow in planta kanamycin selection of transformed roots into a level 2 destination vector (EC50507). For co-expression experiments, *pCEP7:GUS* and *pAtUbi:NIN:GFP* constructs were combined into a single vector. LUC (EC15217), GUS (EC75111), GFP (EC15095), pAtUbi (EC15062), and 35S terminator (EC41414) cassettes were also provided by the ENSA project. For the *CEP7* promoter, a ~2.4-kb region upstream of the predicted ATG was selected, whereas the ~2-kb region of the *MtCLE13* promoter previously characterized by Mortier et al.^28^ was used and amplified by PCR on Jemalong A17 genomic DNA using primers listed in Supplementary Table 1. The same region of the *MtCLE13* promoter was used to express *MtCEP7* using the level 1 vector. The efficiency of this *MtCEP7* ectopic expression construct was evaluated on roots collected 21 dpi. The *pCEP7ΔNBS:GUS* promoter was constructed by combining two PCR products, amplified, respectively, right upstream and downstream of the predicted NBS *cis*-element (primers listed in the Supplementary Table 1). The *pAtUbi:amiRCEP7* amiRNA construct was designed based on the Carbonell et al.^61^ strategy (amiRNA sequence provided in Supplementary Table 1). The amiRCEP7 cassette was synthetized by Genewiz (https://www.genewiz.com/) and then assembled with the *pAtUbi* promoter in the level 1 vector. The efficiency of the amiRCEP7 construct was evaluated on roots collected 2 dpi.

Constructs of interest were then introduced into the *A. rhizogenes* ARqua1 strain and recombinant Arqua1 bacteria were used to transform roots according to the protocol described in Boisson-Dernier et al.^62^. The *A. rhizogenes* strains were grown at 28 °C for 2 days on a YEB-agar medium supplemented with appropriate antibiotics (Duchefa, https://www.duchefa-biochemie.com/). Infected seedlings were grown in vitro on a Fåhraeus medium supplemented with 1 mM NH_4_NO_3_ (Truchet et al.)^54^ and kanamycin (25 mg L^−1^; Duchefa) for 1 week at 20 °C, and 1 more week at 24 °C under a 16-h light photoperiod (150 μE). Plantlets with kanamycin-resistant transformed roots were then transferred either in vitro onto a growth paper (Mega International, http://www.mega-international.com/) on nitrogen-free Fåhraeus medium, grown for 5 days, and then inoculated with rhizobia as previously described or transferred to pots containing a perlite:sand 3:1 mixture for 5 days and then inoculated with rhizobia. Nodule number was scored at 14 dpi for plants grown in vitro or at 21 dpi for plants grown in perlite/sand pots. Images of nodules were obtained in bright field using an AxioZoom V16 macroscope (Zeiss, https://www.zeiss.fr).

### Transient activation assays in *M. truncatula* protoplasts

Leaf protoplasts were isolated from 8-week-old *M. truncatula* plants as described by Yoo et al.^63^, except that 2% Cellulase Onozuka R10 (Yakult Pharmaceutical Industry CO LTD), 1% Macerozyme R10 (Yakult Pharmaceutical Industry CO LTD), and 4% Viscozyme (Sigma) enzymes were used to digest the cell walls. Protoplasts (2.5 × 10^5^) were transfected with 20 μg of a DNA mixture consisting of 9 μg of either the *pCLE13:LUC* or the *pCEP7:LUC* reporter construct, 10 µg of the *p35S-3xHA-NIN* or of the empty vector construct as a negative control, and 1 μg of the *pAtUbi:GUS* vector used as an internal control to normalize the transfection efficiency. After transfection, the protoplasts were incubated overnight at 20 °C in darkness. Protoplasts were then harvested by centrifugation (100 × *g* for 5 min), lysed in a protoplast lysis buffer^63^, and protein extracts were clarified by centrifugation (2000 × *g* for 5 min). For GUS assays, protein extracts were incubated 30 min at 37 °C with the 4-methylumbelliferyl-β-d-glucuronide hydrate (MUG, Duchefa, https://www.duchefa-biochemie.com/) substrate (1 mM 4-MUG in 50 mM sodium phosphate (pH 7.0), 0.1% Triton X-100, 10 mM EDTA, 0.1% sodium lauryl sarcosine, and 10 mM dithiothreitol (DTT)), and fluorescence was measured with an Infinite200 microplate reader (Tecan, http://www.tecan.com). For luciferase (LUC) assays, protein extracts were mixed with a luciferase assay buffer (20 mM Tricine pH 7.8, 5 mM MgCl2, 0.1 mM EDTA, 3.3 mM DTT, 270 µM Coenzyme A, 500 µM Luciferin (Duchefa), and 500 µM ATP), and luminescence was immediately measured for 10 s using the Tecan Infinite200 microplate reader. LUC/GUS ratios were calculated and normalized relative to the empty vector.

### qRT-PCR gene expression analysis

Total RNAs were isolated from frozen roots with the RNeasy Plant Mini Kit (Qiagen, http://www.qiagen.com/) according to the manufacturer’s instructions. Total RNAs (1.5 µg) were used for cDNA synthesis with the Superscript II Reverse Transcriptase (ThermoFisher, http://www.thermofisher.com). qRT-PCR experiments were performed on a LightCycler 480 apparatus using the LightCycler480 SYBR Green I Master Kit (Roche Diagnostics, http://lifescience.roche.com) according to the manufacturer’s instructions. Cycling conditions were as follows: 95 °C for 5 min, and then 40 cycles at 95 °C for 15 s, 60 °C for 15 s, and 72 °C for 15 s. A dissociation curve (55–95 °C) was performed to assess the specificity of the amplification. The expression of genes of interest was normalized against the reference genes *MtACTIN11* and *MtRBP1* (*RNA binding protein 1*) previously selected using the Genorm software (https://genorm.cmgg.be/)^64^. All primers used in qRT-PCR are listed in Supplementary Table 1. Cycle threshold (Ct) values obtained for the two reference genes were averaged prior to calculation of ratios of genes of interest onto reference genes. These ratios were calibrated relative to the experimental control condition (WT genotype and/or untreated mock control).

### ChIP-qPCR assays

ChIP experiments were performed on WT or *nin* roots (~3 g of fresh weight) grown in vitro on a nitrogen-free Fåhraeus medium and inoculated 5 days with rhizobia. Briefly, after plant material fixation in 1% (v/v) formaldehyde for 30 min, roots were frozen and ground in liquid nitrogen; then nuclei were isolated and lysed according to Gendrel et al.^65^. Chromatin was then sonicated using a water bath Bioruptor® Plus sonicator (Diagenode; 40 cycles of 30 s on/30 s off pulses). IPs were performed on the sonicated chromatin using an α-NIN antibody (5:1000; Vernié et al.^47^) or IgG (Sigma Aldrich) as a negative control, as described in Gendrel et al.^65^, except that washes were performed with a lower stringency using a ChIP dilution buffer (167 mM NaCl, 16.7 mM Tris-Hcl pH 8, 1.2 mM EDTA, 1.1% Triton X-100).

Immunoprecipitated DNA was analyzed by qPCR (Roche Diagnostics, http://lifescience.roche.com) as previously described for RT-PCR, using primers listed in Supplementary Table 1. IP/input ratios were calculated and normalized relative to ratios of IgG IPs.

### GUS histochemical staining

GUS activity was detected using histochemical staining as previously described (Pichon et al.^66^; roots were incubated for 3 h at 37 °C). After staining, roots were washed twice in sterile water and observed in bright field using an AxioZoom V16 macroscope (Zeiss, https://www.zeiss.fr) or a Leica DM550 B (Leica Microsystems). Root sections were included in 3% agarose and then sliced into 80-µm sections using a VT 1200S vibratome (Leica Microsystems, http://www.leica-microsystems.com/). Vibratome sections mounted in water were observed in bright field using with an Olympus BX53 microscope (www.olympus-lifescience.com).

### Statistical analyses

At least three independent biological replicates were performed in all experiments. Mann–Whitney non-parametric test, available in the Xlstat software (http://www.xlstat.com/), was used to assess significant differences. To compare proportions of roots showing different *GUS* expression patterns, Fisher exact test was used.

### Accession numbers

Sequence data from this article were retrieved from the *M. truncatula* genome v4.0 (https://phytozome.jgi.doe.gov/; Tang et al.^67^) and/or the *M. truncatula* genome v5 (https://medicago.toulouse.inra.fr/MtrunA17r5.0-ANR/; Pecrix et al.^68^) under the following accession numbers, respectively: *MtCLE13*, Medtr4g079610.1 or MtrunA17Chr4g0040951; *MtCRA2*, Medtr3g110840.1 or MtrunA17Chr3g0140861; *MtCRE1*, Medtr8g106150.1 or MtrunA17Chr8g0392301, *MtNIN*, Medtr5g099060 or MtrunA17Chr5g0448621; *MtCEP7*, MtrunA17Chr8g0374811. The *MtCEP7* sequence, absent from the v4.0 version, was alternatively retrieved from the *M. truncatula* v3.5 database (AC233112_1013; http://blast.jcvi.org/Medicago-Blast).

### Reporting summary

Further information on research design is available in the Nature Research Reporting Summary linked to this article.

## Supplementary information

Supplementary Information

Reporting Summary

## Source data

Source Data

## Data Availability

Data supporting the findings of this work are available within the article and its Supplementary Information file. The source data underlying Figs. 1a–c, 2a–i, 3i, 4c–f, 5c, e, and 6c, e, f, h, i, as well as Supplementary Figs. 1, 4A, B, 5A–D, and 6B are provided as a Source data file. Other supporting data or biological materials are available from the corresponding author upon request.

## References

[1] Sprent, J. I. & James, E. K. Legume evolution: where do nodules and mycorrhizas fit in? *Plant Physiol.* **144**, 575–581 (2007). 17556520 10.1104/pp.107.096156 PMC1914177

[2] Suzaki, T., Yoro, E. & Kawaguchi, M. Leguminous plants: inventors of root nodules to accommodate symbiotic bacteria. *Int. Rev. Cell Mol. Biol.* **316**, 111–158 (2015). 25805123 10.1016/bs.ircmb.2015.01.004

[3] Mohd-Radzman, N. A. et al. Different pathways act downstream of the peptide receptor CRA2 to regulate lateral root and nodule development. *Plant Physiol.* **171**, 2536–2548 (2016). 27342310 10.1104/pp.16.00113 PMC4972263

[4] Bhuvaneswari, T. V., Bhagwat, A. A. & Bauer, W. D. Transient susceptibility of root cells in four common legumes to nodulation by Rhizobia. *Plant Physiol.* **68**, 1144–1149 (1981). 16662065 10.1104/pp.68.5.1144 PMC426059

[5] Timmers, A. C. J., Auriac, M. & Truchet, G. Refined analysis of early symbiotic steps of the Rhizobium-Medicago interaction in relationship with microtubular cytoskeleton rearrangements. *Development* **126**, 3617–3628 (1999). 10409507 10.1242/dev.126.16.3617

[6] Xiao, T. T. et al. Fate map of *Medicago truncatula* root nodules. *Development* **141**, 3517–3528 (2014). 25183870 10.1242/dev.110775

[7] Journet, E. et al. *Medicago truncatula* ENOD11: a novel RPRP-encoding early nodulin gene expressed during mycorrhization in arbuscule-containing cells. *Mol. Plant Microbe Interact.* **14**, 737–748 (2001). 11386369 10.1094/MPMI.2001.14.6.737

[8] Andriankaja, A. et al. AP2-ERF transcription factors mediate Nod factor–dependent Mt ENOD11 activation in root hairs via a novel cis-regulatory motif. *Plant Cell* **19**, 2866–2885 (2007). 17827349 10.1105/tpc.107.052944 PMC2048698

[9] Gonzalez-Rizzo, S., Crespi, M. & Frugier, F. The *Medicago truncatula* CRE1 cytokinin receptor regulates lateral root development and early symbiotic interaction with *Sinorhizobium meliloti*. *Plant Cell* **18**, 2680–2693 (2006). 17028204 10.1105/tpc.106.043778 PMC1626621

[10] Murray, J. D. et al. A cytokinin perception mutant colonized by Rhizobium in the absence of nodule organogenesis. *Science* **315**, 101–104 (2007). 17110535 10.1126/science.1132514

[11] Plet, J. et al. MtCRE1-dependent cytokinin signaling integrates bacterial and plant cues to coordinate symbiotic nodule organogenesis in *Medicago truncatula*. *Plant J.* **65**, 622–633 (2011). 21244535 10.1111/j.1365-313X.2010.04447.x

[12] Van Zeijl, A. et al. Rhizobium lipo-chitooligosaccharide signaling triggers accumulation of cytokinins in *Medicago truncatula* roots. *Mol. Plant* **8**, 1213–1226 (2015). 25804975 10.1016/j.molp.2015.03.010

[13] Gamas, P., Brault, M., Jardinaud, M. & Frugier, F. Cytokinins in symbiotic nodulation: when, where, what for? *Trends Plant Sci.* **22**, 792–802 (2017). 28739135 10.1016/j.tplants.2017.06.012

[14] Tirichine, L. et al. A gain-of-function mutation in a root nodule organogenesis. *Science* **2680**, 104–107 (2007). 10.1126/science.1132397 17110537

[15] Jardinaud, M.-F. et al. A laser dissection-RNAseq analysis highlights the activation of cytokinin pathways by Nod factors in the *Medicago truncatula* root epidermis. *Plant Physiol.* **171**, 2256–2276 (2016). 27217496 10.1104/pp.16.00711 PMC4936592

[16] Tsikou, D. et al. Systemic control of legume susceptibility to rhizobial infection by a mobile microRNA. *Science* **362**, 233–236 (2018). 30166437 10.1126/science.aat6907

[17] Miri, M. et al. Inside out: root cortex-localized LHK1 cytokinin receptor limits epidermal infection of Lotus japonicus roots by *Mesorhizobium loti*. *N. Phytol.* **222**, 1523–1537 (2019). 10.1111/nph.15683 30636324

[18] Schauser, L., Roussis, A., Stiller, J. & Stougaard, J. A plant regulator controlling development of symbiotic root nodules. *Nature* **402**, 191–195 (1999). 10647012 10.1038/46058

[19] Middleton, P. H. et al. An ERF transcription factor in *Medicago truncatula* that is essential for Nod factor signal transduction. *Plant Cell* **19**, 1221–1234 (2007). 17449807 10.1105/tpc.106.048264 PMC1913751

[20] Liu, C.-W. et al. NIN acts as a network hub controlling a growth module required for rhizobial infection. *Plant Physiol.* **179**, 1704–1722 (2019). 30710053 10.1104/pp.18.01572 PMC6446755

[21] Fournier, J. et al. Remodeling of the infection chamber before infection thread formation reveals a two-step mechanism for rhizobial entry into the host legume root hair. *Plant Physiol.* **167**, 1233–1242 (2015). 25659382 10.1104/pp.114.253302 PMC4378154

[22] Soyano, T., Kouchi, H., Hirota, A. & Hayashi, M. NODULE INCEPTION directly targets NF-Y subunit genes to regulate essential processes of root nodule development in *Lotus japonicus*. *PLoS Genet.* **9**, e1003352 (2013). 23555278 10.1371/journal.pgen.1003352 PMC3605141

[23] Marsh, J. F. et al. *Medicago truncatula* NIN is essential for rhizobial-independent nodule organogenesis induced by autoactive calcium/calmodulin-dependent protein kinase 1. *Plant Physiol.* **144**, 324–335 (2007). 17369436 10.1104/pp.106.093021 PMC1913781

[24] Yoro, E. et al. A positive regulator of nodule organogenesis, NODULE INCEPTION, acts as a negative regulator of rhizobial infection in *Lotus japonicus*. *Plant Physiol.* **165**, 747–758 (2014). 24722550 10.1104/pp.113.233379 PMC4043699

[25] Liu, J. et al. A remote cis-regulatory region is required for NIN expression in the pericycle to initiate nodule primordium formation in *Medicago truncatula*. *Plant Cell* **31**, 68–83 (2019). 30610167 10.1105/tpc.18.00478 PMC6391699

[26] Caetano-Anolles, G. & Gresshoff, P. M. Plant genetic control of nodulation. *Annu. Rev. Microbiol.* **45**, 345–382 (1991). 1741618 10.1146/annurev.mi.45.100191.002021

[27] Okamoto, S. et al. Nod factor/nitrate-induced CLE genes that drive HAR1-mediated systemic regulation of nodulation. *Plant Cell Physiol.* **50**, 67–77 (2009). 19074184 10.1093/pcp/pcn194

[28] Mortier, V. et al. CLE peptides control *Medicago truncatula* nodulation locally and systemically. *Plant Physiol.* **153**, 222–237 (2010). 20348212 10.1104/pp.110.153718 PMC2862434

[29] Reid, D. E., Ferguson, B. J. & Gresshoff, P. M. Inoculation- and nitrate-induced CLE peptides of soybean control NARK-dependent nodule formation. *Mol. Plant Microbe Interact.* **24**, 606–618 (2011). 21198362 10.1094/MPMI-09-10-0207

[30] Nishida, H., Handa, Y., Tanaka, S. & Suzaki, T. Expression of the CLE ‑ RS3 gene suppresses root nodulation in Lotus japonicus. *J. Plant Res.* **129**, 909–919 (2016). 27294965 10.1007/s10265-016-0842-z

[31] Wang, L. et al. A GmNINa-miR172c-NNC1 regulatory network coordinates the nodulation and autoregulation of nodulation pathways in soybean. *Mol. Plant* **12**, 1211–1226 (2019). 31201867 10.1016/j.molp.2019.06.002

[32] Soyano, T., Hirakawa, H., Sato, S., Hayashi, M. & Kawaguchi, M. NODULE INCEPTION creates a long-distance negative feedback loop involved in homeostatic regulation of nodule organ production. *Proc. Natl Acad. Sci. USA* **111**, 14607–14612 (2014). 25246578 10.1073/pnas.1412716111 PMC4210044

[33] Krusell, L. et al. Shoot control of root development and nodulation is mediated by a receptor-like kinase. *Nature* **420**, 422–426 (2002). 12442170 10.1038/nature01207

[34] Searle, I. R. et al. Long-distance signaling in nodulation directed by a CLAVATA1-like receptor kinase. *Science* **299**, 109–112 (2003). 12411574 10.1126/science.1077937

[35] Schnabel, E., Journet, E. P., De Carvalho-Niebel, F., Duc, G. & Frugoli, J. The *Medicago truncatula* SUNN gene encodes a CLV1-like leucine-rich repeat receptor kinase that regulates nodule number and root length. *Plant Mol. Biol.* **58**, 809–822 (2005). 16240175 10.1007/s11103-005-8102-y

[36] Imin, N., Patel, N., Corcilius, L. & Payne, R. J. CLE peptide tri-arabinosylation and peptide domain sequence composition are essential for SUNN-dependent autoregulation of nodulation in *Medicago truncatula*. *N. Phytol.* **218**, 73–80 (2018). 10.1111/nph.15019 29393515

[37] Gautrat, P., Laffont, C. & Frugier, F. Compact root architecture 2 promotes root competence for nodulation through the miR2111 systemic effector. *Curr. Biol.* **30**, 1–7 (2020). 32109394 10.1016/j.cub.2020.01.084

[38] Imin, N., Mohd-Radzman, N. A., Ogilvie, H. A. & Djordjevic, M. A. The peptide-encoding CEP1 gene modulates lateral root and nodule numbers in *Medicago truncatula*. *J. Exp. Bot.* **64**, 5395–5409 (2013). 24259455 10.1093/jxb/ert369

[39] Huault, E. et al. Local and systemic regulation of plant root system architecture and symbiotic nodulation by a receptor-like kinase. *PLoS Genet.* **10**, e1004891 (2014). 25521478 10.1371/journal.pgen.1004891 PMC4270686

[40] Mohd-Radzman, N. A., Binos, S., Truong, T. T. & Djordjevic, M. A. Novel MtCEP1 peptides produced in vivo differentially regulate root development in *Medicago truncatula*. *J. Exp. Bot.* **66**, 5289–5300 (2015). 25711701 10.1093/jxb/erv008 PMC4526912

[41] Laffont, C. et al. Independent regulation of symbiotic nodulation by the SUNN negative and CRA2 positive systemic pathways. *Plant Physiol.* **180**, 559–570 (2019). 30782966 10.1104/pp.18.01588 PMC6501087

[42] Delay, C., Imin, N. & Djordjevic, M. A. CEP genes regulate root and shoot development in response to environmental cues and are specific to seed plants. *J. Exp. Bot.* **64**, 5383–5394 (2013). 24179096 10.1093/jxb/ert332

[43] Tabata, R. et al. Perception of root-derived peptides by shoot LRR-RKs mediates systemic N-demand signaling. *Science* **346**, 343–346 (2014). 25324386 10.1126/science.1257800

[44] Chapman, K., Taleski, M., Ogilvie, H. A., Imin, N. & Djordjevic, M. A. CEP–CEPR1 signalling inhibits the sucrose-dependent enhancement of lateral root growth. *J. Exp. Bot.* **70**, 3955–3967 (2019). 31056646 10.1093/jxb/erz207 PMC6685651

[45] Amor, B. Ben et al. The NFP locus of *Medicago truncatula* controls an early step of Nod factor signal transduction upstream of a rapid calcium flux and root hair deformation. *Plant J.* **34**, 495–506 (2003). 12753588 10.1046/j.1365-313x.2003.01743.x

[46] Mortier, V., De Wever, E., Vuylsteke, M., Holsters, M. & Goormachtig, S. Nodule numbers are governed by interaction between CLE peptides and cytokinin signaling. *Plant J.* **70**, 367–376 (2012). 22168914 10.1111/j.1365-313X.2011.04881.x

[47] Vernié, T. et al. The NIN transcription factor coordinates diverse nodulation programs in different tissues of the *Medicago truncatula* root. *Plant Cell* **27**, 3410–3424 (2015). 26672071 10.1105/tpc.15.00461 PMC4707452

[48] Ogilvie, H. A., Imin, N. & Djordjevic, M. A. Diversification of the C-TERMINALLY ENCODED PEPTIDE (CEP) gene family in angiosperms, and evolution of plant-family specific CEP genes. *BMC Genomics* **15**, 6–10 (2014). 25287121 10.1186/1471-2164-15-870 PMC4197245

[49] Whitewoods, C. D. et al. CLAVATA was a genetic novelty for the morphological innovation of 3D growth in land plants. *Curr. Biol.* **28**, 2365–2376 (2018). 30033333 10.1016/j.cub.2018.05.068 PMC6089843

[50] Chapman, K. et al. CEP receptor signalling controls root system architecture in Arabidopsis and Medicago. *N. Phytol.*10.1111/nph.16483 (2020). 10.1111/nph.16483 32048296

[51] Oh, E., Seo, P. J. & Kim, J. Signaling peptides and receptors coordinating plant root development. *Trends Plant Sci.* **23**, 337–351 (2018). 29366684 10.1016/j.tplants.2017.12.007

[52] Cammarata, J., Roeder, A. H. & Scanlon, M. J. Cytokinin and CLE signaling are highly intertwined developmental regulators across tissues and species. *Curr. Opin. Plant Biol.* **51**, 96–104 (2019). 31280129 10.1016/j.pbi.2019.05.006

[53] Yadav, R. K. et al. WUSCHEL protein movement mediates stem cell homeostasis in the Arabidopsis shoot apex. *Genes Dev.* **25**, 2025–2030 (2011). 21979915 10.1101/gad.17258511 PMC3197201

[54] Truchet, G. et al. Alfalfa nodulation in the absence of Rhizobium. *Mol. Gen. Genet.* **219**, 65–68 (1989).

[55] Blondon, F. Contribution à l’étude du développement des graminées fourragères ray-grass et dactyle. *Rev. Gen. Bot.* **71**, 293–381 (1964).

[56] Ardourel, M. et al. *Rhízobíum meliloti* lipooligosaccharide nodulation factors: different structural requirements for bacterial entry into target root hair cells and lnduction of plant symbiotic developmental responses. *Plant Cell* **6**, 1357–1374 (1994). 7994171 10.1105/tpc.6.10.1357 PMC160526

[57] Vervliet, G., Holsters, M., Teuchy, H., Van Montagu, M. & Schell, J. Characterization of different plaque-forming and defective temperate phages in Agrobacterium. *J. Gen. Virol.* **26**, 33–48 (1975). 1123610 10.1099/0022-1317-26-1-33

[58] Karimi, M., Inzé, D. & Depicker, A. GATEWAY^TM^ vectors for Agrobacterium-mediated plant transformation. *Trends Plant Sci.* **7**, 1–3 (2002). 11992820 10.1016/s1360-1385(02)02251-3

[59] Engler, C. & Marillonnet, S. Golden Gate cloning. *Methods Mol. Biol.* **1116**, 119–131 (2014). 24395361 10.1007/978-1-62703-764-8_9

[60] Weber, E., Engler, C., Gruetzner, R., Werner, S. & Marillonnet, S. A modular cloning system for standardized assembly of multigene constructs. *PLoS ONE* **6**, e16765 (2011). 21364738 10.1371/journal.pone.0016765 PMC3041749

[61] Carbonell, A. et al. New generation of artificial microRNA and synthetic trans-acting small interfering RNA vectors for efficient gene silencing in Arabidopsis. *Plant Physiol.* **165**, 15–29 (2014). 24647477 10.1104/pp.113.234989 PMC4012576

[62] Boisson-Dernier, A. et al. *Agrobacterium rhizogenes* -transformed roots of *Medicago truncatula* for the study of nitrogen-fixing and endomycorrhizal symbiotic associations. *Mol. Plant Microbe Interact.* **14**, 695–700 (2001). 11386364 10.1094/MPMI.2001.14.6.695

[63] Yoo, S.-D., Cho, Y.-H. & Sheen, J. Arabidopsis mesophyll protoplasts: a versatile cell system for transient gene expression analysis. *Nat. Protoc.* **2**, 1565–1572 (2007). 17585298 10.1038/nprot.2007.199

[64] Vandesompele, J. et al. Accurate normalization of real-time quantitative RT -PCR data by geometric averaging of multiple internal control genes. *Genome Biol.* **3**, 1–12 (2002). 10.1186/gb-2002-3-7-research0034 PMC126239 12184808

[65] Gendrel, A. V., Lippman, Z., Martienssen, R. & Colot, V. Profiling histone modification patterns in plants using genomic tiling microarrays. *Nat. Methods* **2**, 213–218 (2005). 16163802 10.1038/nmeth0305-213

[66] Pichon, M. et al. *Rhizobium meliloti* elicits transient expression of the early nodulin gene ENOD12 in the differentiating root epidermisof transgenic alfalfa. *Plant Cell* **4**, 1199–1211 (1992). 1446169 10.1105/tpc.4.10.1199 PMC160208

[67] Tang, H. et al. An improved genome release (version Mt4. 0) for the model legume *Medicago truncatula*. *BMC Genomics* **15**, 1–14 (2014). 24767513 10.1186/1471-2164-15-312 PMC4234490

[68] Pecrix, Y. et al. Whole-genome landscape of *Medicago truncatula* symbiotic genes. *Nat. Plants* **4**, 1017–1025 (2018). 30397259 10.1038/s41477-018-0286-7

