# Supplementary material for: The NIN transcription factor coordinates CEP and CLE signaling peptides that regulate nodulation antagonistically
Source: Nat Commun. 2020 Jun 23;11:3167. doi: 10.1038/s41467-020-16968-1 (PMC7311451; doi:10.1038/s41467-020-16968-1)
Supplement: Supplementary file 1 — Supplementary Information [file 41467_2020_16968_MOESM1_ESM.pdf]

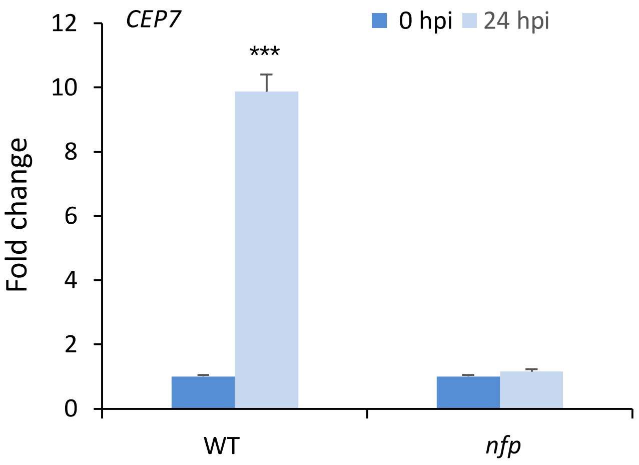

Supplementary Figure 1. The induction of *CEP7* expression by rhizobium relies on NFP
Expression analysis of the *MtCEP7* gene in Wild-Type (WT) or *nfp* (*nod factor perception*) mutant roots 24 hours post rhizobium inoculation (hpi). qRT-PCR was used to measure gene expression levels in treated roots, normalized relative to untreated roots. Error bars represent confidence intervals (α=0.05) from three biological replicates (n=9).

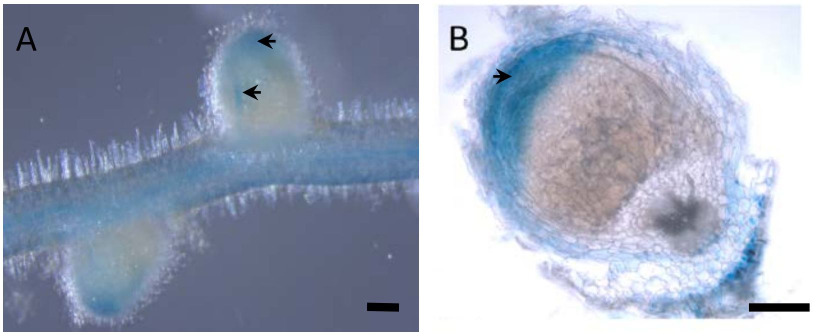

Supplementary Figure 2. Rhizobia inoculation induces a *pMtCEP7:GUS* transcriptional fusion in vascular bundles and the apex of mature nodules

A-B. The *pCEP7:GUS* activity was analyzed in mature nodules 14 days post rhizobium inoculation (dpi). A is a whole mount image, and B is a sagittal root and nodule section.

A minimum of 8 independent roots were analyzed for each condition from five independent experiments. Images show the GUS staining as a blue signal in bright field microscopy, indicated with arrows. Scale bars=200 µm.

A

MGLFQVTTKYLIVILALSIVYNSFQITQARPIKPLNQQSSLNTQDSGAIHTNSFRPTTPGSSPGVGHRNFVVGDKNTR
TMVVVQSPDVEVFVTNKRSDDGFKPTNPSHSPGVGHGYHTKIRHLN*

B

GGGCATTGAACTCAATACAAAGATTATAAATAGCATAAGACCCCAGAGCTTGTGTTATAATTTTCTTTTCCATTG
CCAATATATAGCTTAATCGTTCTCTCTAGTTTTAACCTACATATATCCTTTCACTCACTTACCTTTTGTTTGTATAG
*TC*ATGGGTTTGTTTCAAGTCACAACAAAATATTTGATCGTTATTCTAGCACTAAGTATTGTATACAATTCCTTTCA
AATAACTCAAGCCAGGCCAATTAAACCATTGAATCAACAATCTTCATTAAACACACAAGACTCGGGTGCAATCC
ACACTAACTCTTTTCGGCCGACAACACCAGGAAGTAGTCCTGGTGTTGGCCACCGAAATTTTGTTGTAGGAGAT
AAGAACACGAGAACAATGGTGGTTGTTCAGAGCCCGGATGTTGAGGTTTTTGTGACGAATAAGAGATCCGAT
GATGGTTTCAAACCTACAAATCCTAGTCATAGTCCTGGAGTTGGCCATGGTTACCATACCAAAATTAGACATTT
AAATTAG*AACGTGATTGAATTCATTGTAGATTTCAAAACCATCTTAATTAGAAGCTGTGTGTACCTACAATAATG*
TCCTTAGTTTCATTCTTCAAATTGCATGCCATATCATATGCATGTAGCACATGCACTTGGACTTTTGTTATTTGTTT
AAGAGGCTTGGCTTTTGGTTATTGTTTGGGGGTGTCATAATTCAAAGTATGTAAAGTTTACTCTATTTATTTGTA
ATTGTTTGGTTAATATGATACAAATCATAGCAAAATAAATAAAAGATGTTTTAG

Supplementary Figure 3. *CEP7* protein and genomic sequences
A. MtCEP7 precursor sequence highlighting the CEP domains 1 (CEP7 D1, in blue) and 2 (CEP7 D2, in green).
B. *CEP7* genomic sequence highlighting regions used for RNA interference (in blue) and artificial microRNA (in yellow) strategies. Italics indicate predicted 5’ and 3’ untranslated regions (UTRs), and initiation and stop codons are underlined.

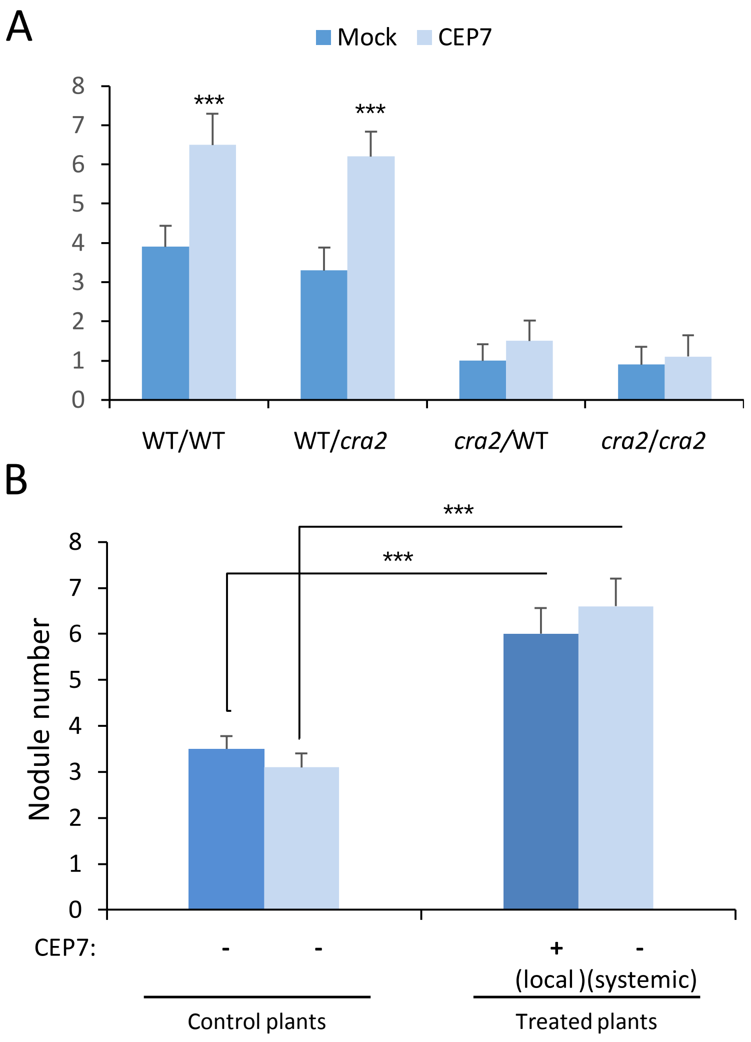

Supplementary Figure 4. CEP7 promotes nodulation systemically

A. Nodulation phenotype of grafts between Wild-Type (WT) and *cra2* mutants treated or not with synthetic MtCEP7 peptides. Quantification of the nodule number in different combinations of WT and *cra2* mutant grafted plants, grown *in vitro* on a nitrogen-free Fahraeus medium supplemented or not with 1 µM CEP7 Domain1 (CEP7 D1) peptides. Nodule number was scored at 14 days post rhizobium inoculation (dpi). Error bars represent confidence intervals (α=0.05) from one representative biological experiment out of two (n>9 plants for each biological replicate). A Mann-Whitney test was used for each graft combination to assess significant differences between peptide-treated plants and the control, as indicated by asterisks (*** α<0.001).

B. Nodulation phenotype of local and systemic roots from plants grown in a split-root experimental system. Quantification of the nodule number in split-root WT plants, grown *in vitro* on a nitrogen-free Fahraeus medium, where half of the root system was treated, or not, with 1 µM CEP7 Domain1 (CEP7 D1) peptides. Rhizobium inoculation was performed on the whole root system. Nodule number was scored at 14 days post-rhizobium inoculation (dpi). Error bars represent confidence intervals (α=0.05) from one representative biological experiment out of two (n > 10 plants for each biological replicate). A Mann-Whitney test was used for each split root combination to assess significant differences between local and systemic roots for peptide-treated plants and the control, as indicated by asterisks (*** α<0.001).

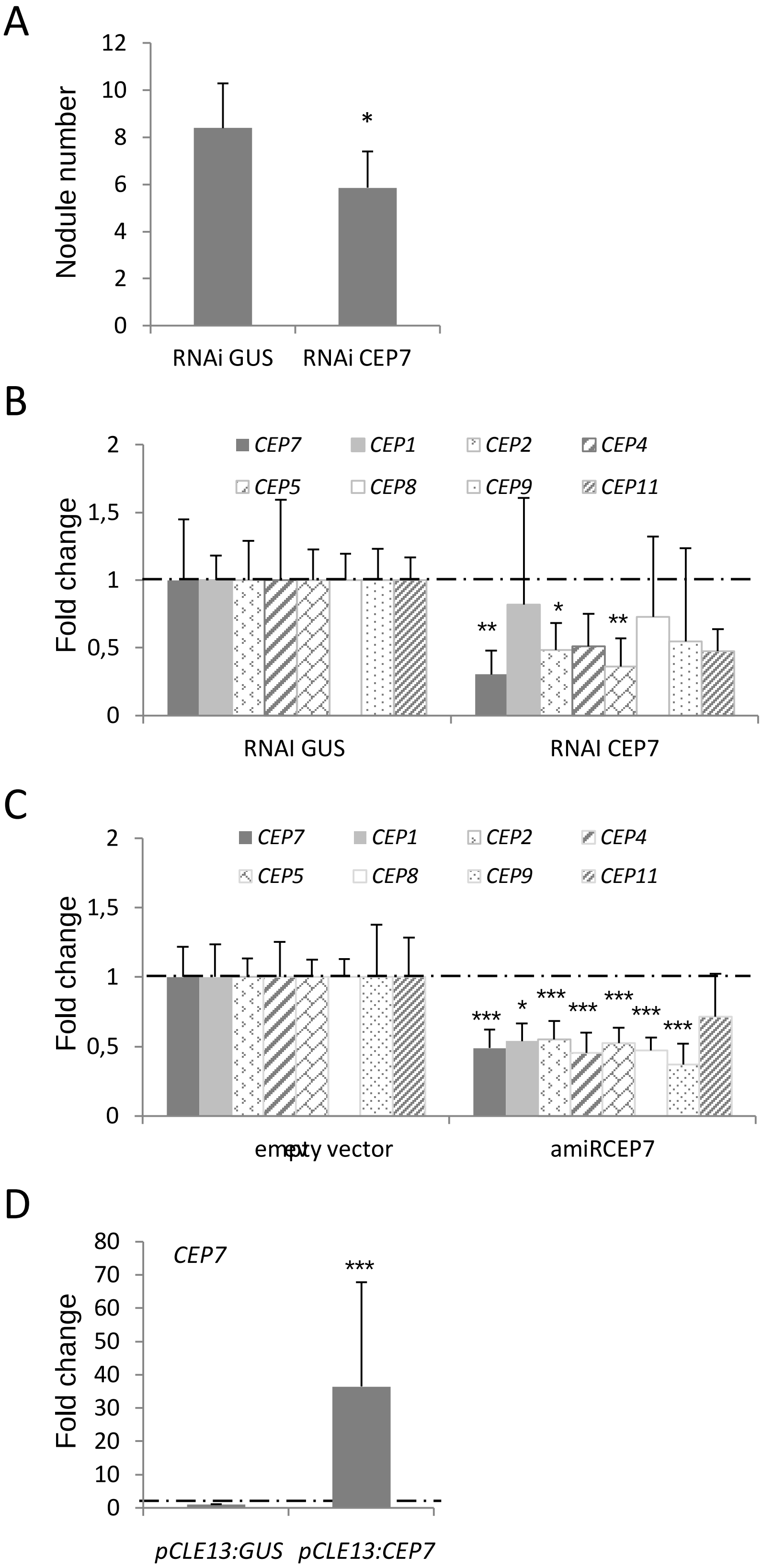

Supplementary Figure 5. Efficiency and specificity of RNA interference, artificial microRNA and CEP7 ectopic expression

A. Nodulation phenotype of plants where *MtCEP7* expression was silenced by RNA interference (RNAi).

Quantification of the nodule number of Wild-Type (WT) plants expressing a RNAi construct driven by the 35S:CaMV promoter and targeting either *GUS* as a negative control or the *MtCEP7* gene. Plants were grown *in vitro* on a nitrogen-free Fahraeus medium and the nodules were scored at 14 days post rhizobium inoculation (dpi). Error bars represent confidence intervals (α=0.05) from one representative biological replicate out of two (n>13 plants for each biological replicate). A Mann-Whitney test was used to assess significant differences, as indicated by asterisks (α<0.05).

B. Expression analysis by qRT-PCR of *CEP* genes in roots expressing a *MtCEP7* RNAi or a *GUS* RNAi construct, as a negative control, grown as described in A. The expression data were normalized relative to the *GUS* control. The dotted line corresponds to a ratio of 1 to highlight fold changes. Error bars represent confidence intervals (α=0.05) from three biological replicates (n=6).

C. Expression analysis by qRT-PCR of *CEP* genes in roots transformed either with an empty vector or expressing an amiRCEP7 construct, grown as described in Figure 6D-F. Expression levels in amiRCEP7 roots were normalized relative to control roots. The dotted line corresponds to a ratio of 1 to highlight fold changes. Error bars represent confidence intervals (α=0.05) from six biological replicates (n=12).

D. Expression analysis by qRT-PCR of *MtCEP7* in roots transformed either with a *pCLE13:GUS* or a *pCLE13:CEP7* construct, grown as described in Figure 6G-I. Expression levels were normalized relative to *pCLE13:GUS* control roots. The dotted line corresponds to a ratio of 1 to highlight fold changes. Error bars represent confidence intervals (α=0.05) from four biological replicates (n=8)

In B-D, a Mann-Whitney test was used to assess significant differences, as indicated by asterisks (* α<0.05; ** α<0.01; *** α<0.001).

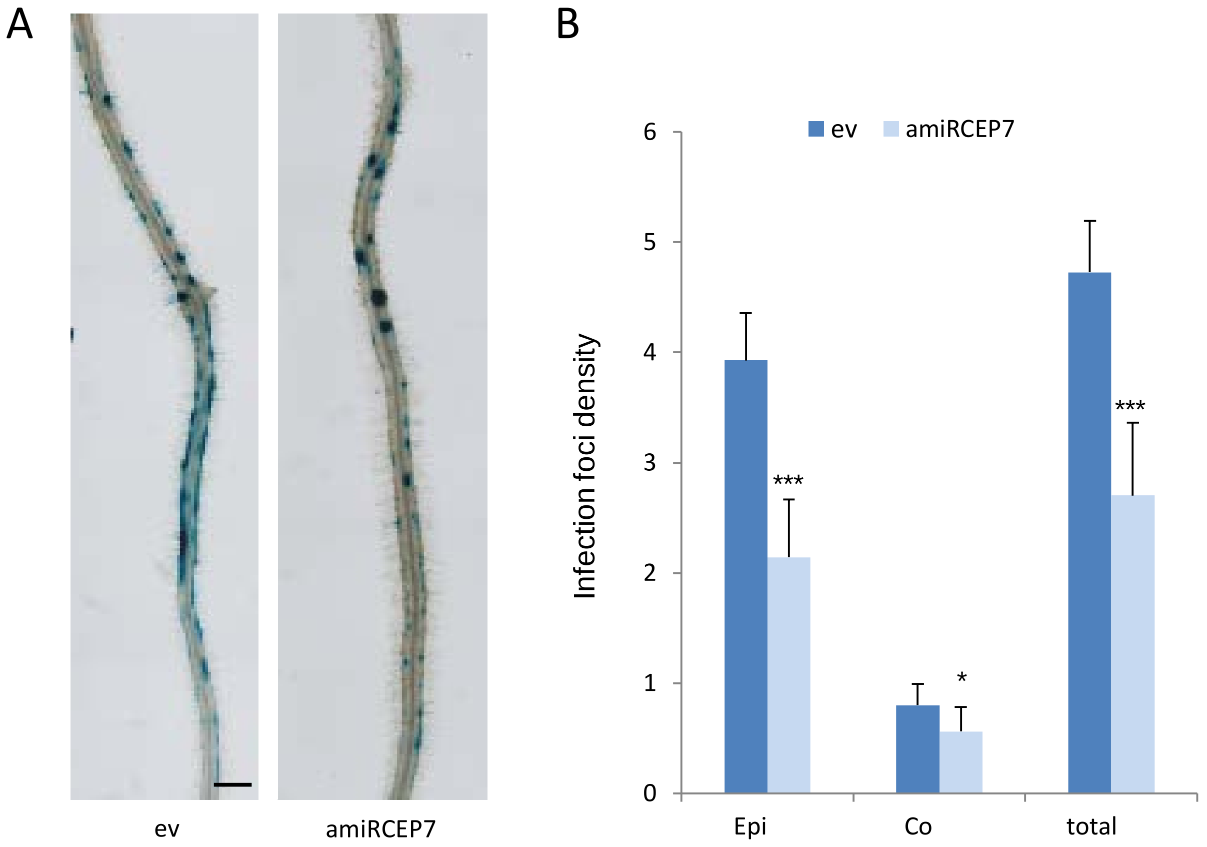

Supplementary Figure 6. The amiRCEP7 construct decreases rhizobial infections

A. Histochemical GUS staining of *pENOD11:GUS* roots five days post rhizobium inoculation (dpi), expressing either the empty vector or the amiRCEP7 construct. Bars=500 µm.

B. Quantification using the *pENOD11:GUS* reporter shown in A of the density of rhizobial infection foci localized either in the root epidermis (Epi) or in the cortex (Co). The number of foci is normalized relative to the length (in mm) of the root region containing blue-stained foci. Error bars represent confidence intervals ($\alpha=0.05$) from two biological replicates (n>22 plants). A Mann-Whitney test was used for each category to assess significant differences between amiRCEP7 roots and the control, as indicated by asterisks (* $\alpha<0.05$; ***$\alpha<0.001$).

ev, empty vector.

Use	Gene / Construct	Forward Primer (5' à 3')	Reverse Primer (5' à 3')
cloning	RNAi CEP7, with **attB sites** for Gateway cloning	**GGGGACAAGTTTGTACAAAAAAGCAGGCT**GCATGAAAGAACACGAGAACAATGG	**GGGGACCACTTTGTACAAGAAAGCTGGGT**CATGGCATGCAATTTGA
	CEP7 promoter	CACTCTGTGGTCTCAGGAGATATATAAACCATCTTACT	CACTTCGTGGTCTCACATTCATGACTATACAAACAAAAGG
	CEP7 ΔNBS promoter	CACTCTGTGGTCTCATACTGTTATTTGACTTGTACACA	CACTTCGTGGTCTCAAGTACTTCTCTTTAAAATTTGACC
	CLE13 promoter	CACTCTGTGGTCTCAGGAGTCATTCCTAGTAGAACGGCGCC	CACTTCGTGGTCTCACATTGTGTGATTTTAATCTTTGAATGG
	amiRCEP7 synthetic sequence with Bsa adapters for goldengate cloning	CACTCTGTGGTCTCAAATGCTATAGGGGGGAAAAAAAGGTAGTCATCAGATATATATTTTGGTAAGAAAATATAGAAATGAATAATTTCACGTTTAACGAAGAGGAGATGACGTGTGTT<br>CCTTCGAACCCGAGTTTTGTTCGTCTATAAATAGCACCTTCTCTTCTCCTTCTTCCTCACTTCCATCTTTTTAGCTTCACTATCTCTCTATAATCGGTTTTATCTTTCTCTAAGTCACAACCC<br>AAAAAAACAAAGTAGAGAAGAATCTGTAAGAAACCTGTATTGTTCTCGTGTTCTTATCTGATGATGATCACATTCGTTATCTATTTTTTCAGATAAGAAAACGAGAACAAGGTTTCACAT<br>TGGCTCTTCTTACTACAATGAAAAAGGCCGAGGCAAAACGCCTAAAATCACTTGAGAATCAATTCTTTTTACTGTCCATTTAAGCTATCTTTTATAAACGTGTCTTATTTTCTATCTCTTTT<br>GTTTAAACTAAGAAACTATAGTATTTTGTCTAAAACAAAACATGAAAGAACAGATTAGATCTCATCTTTAGTCTCAGCTTTGAGACCACGAAGTG	
qPCR	*MtCEP1*	TGCAAGGCTCATTAAACCATCG	TGGAATGCATTTGTGTGAGCTT
	MtCEP2	TAGCTCGCATTTGCTTGTTC	GGCTGAATGCTTTGTCTCAA
	MtCEP4	TGAAGCATCAAGGTTGCTAAAT	TGAAGGAGCTTGTGGACTTTTA
	MtCEP5	GTGTTGTTTTGAGCCCAAGG	TGTTGGTCGAAAAGCTTCAA
	MtCEP6	GCTCATCATGGAGGGAAGTC	TATGCCCTGGAGATGTAGGC
	MtCEP7	CCGGATGTTGAGGTTTTTGT	GGCCAACTCCAGGACTATGA
	MtCEP7 specifically for the RNAi construct	CTCAAGCCAGGCCAATTAAA	CAACCACCATTGTTCTCGTG
	MtCEP8	TCCAACAATATTGCCACCAA	GGGTTGTGGGTCTAAAAGCA
	MtCEP9	TGATGCCAAATCATGGTGTC	GGACTGCTTCCTGGTGTTGT
	MtCEP11	AGCTCCTTCCATTGGCTTTT	CCCCACCAGGACTATGACC
	MtNIN	GCAATGTGGGGATTTAGAGATT	GGAAGATTGAGAGGGGAAGCTT
	MtCLE13	CCGAAGCCTTCTACAGAAACTACG	TCTTGGTGGTGATCTTCCATTATGC
	MtACTIN11	TGGCATCACTCAGTACCTTTCAACAG	ACCCAAAGCATCAAATAATAAGTCAACC
	MtRBP1	AGGGGCAAGTTCCTTCATTT	AAACGGACGGAAAATGTGAG
	GUS	CTCATCTGGAATTTCGCCGA	GGCGAGTGAAGATCCCCTTC
ChIP-qPCR	pCEP7 q1	CTGCAGGGTCCATTTTGATT	AGTTGTTGCAAGTCCGATCC
	pCEP7 q2	ACACACCAACGGCTCACAT	CAAGCTCTGGGGTCTTATGC
	pCLE13 q1	GAGCTTCTGAAGGAGGCACA	TGTCGCCATTTTTGTGGATA
	pCLE13 q2	GGTCACATGCTCTGGGATTT	TGATGGACGGTGCTTAAGGT

Supplementary Table 1. List of primers used and sequence of the MtCEP7 artificial microRNA construct

Primers used for cloning, qRT-PCR and ChIP-qPCR experiments are listed, as well as the sequence of the MtCEP7 artificial microRNA construct.

Adapters for Golden Gate are underlined, adapters for Gateway cloning are in bold.
